# Insights on Some Polysaccharide Gel Type Materials and Their Structural Peculiarities

**DOI:** 10.3390/gels8120771

**Published:** 2022-11-26

**Authors:** Ioana Alexandra Duceac, Magdalena-Cristina Stanciu, Marioara Nechifor, Fulga Tanasă, Carmen-Alice Teacă

**Affiliations:** 1 Polyaddition and Photochemistry Department, “Petru Poni” Institute of Macromolecular Chemistry, 41A Gr. Ghica-Voda Alley, 700487 Iasi, Romania; 2 Natural Polymers, Bioactive and Biocompatible Materials Department, “Petru Poni” Institute of Macromolecular Chemistry, 41A Gr. Ghica-Voda Alley, 700487 Iasi, Romania; 3 Center for Advanced Research in Bionanoconjugates and Biopolymers, “Petru Poni” Institute of Macromolecular Chemistry, 41A Gr. Ghica-Voda Alley, 700487 Iasi, Romania

**Keywords:** polysaccharides, gel type materials, chitosan, dextran, starch, alginate, cellulose, structure, properties

## Abstract

Global resources have to be used in responsible ways to ensure the world’s future need for advanced materials. Ecologically friendly functional materials based on biopolymers can be successfully obtained from renewable resources, and the most prominent example is cellulose, the well-known most abundant polysaccharide which is usually isolated from highly available biomass (wood and wooden waste, annual plants, cotton, etc.). Many other polysaccharides originating from various natural resources (plants, insects, algae, bacteria) proved to be valuable and versatile starting biopolymers for a wide array of materials with tunable properties, able to respond to different societal demands. Polysaccharides properties vary depending on various factors (origin, harvesting, storage and transportation, strategy of further modification), but they can be processed into materials with high added value, as in the case of gels. Modern approaches have been employed to prepare (e.g., the use of ionic liquids as “green solvents”) and characterize (NMR and FTIR spectroscopy, X ray diffraction spectrometry, DSC, electronic and atomic force microscopy, optical rotation, circular dichroism, rheological investigations, computer modelling and optimization) polysaccharide gels. In the present paper, some of the most widely used polysaccharide gels will be briefly reviewed with emphasis on their structural peculiarities under various conditions.

## 1. Introduction

**Scheme 1:**
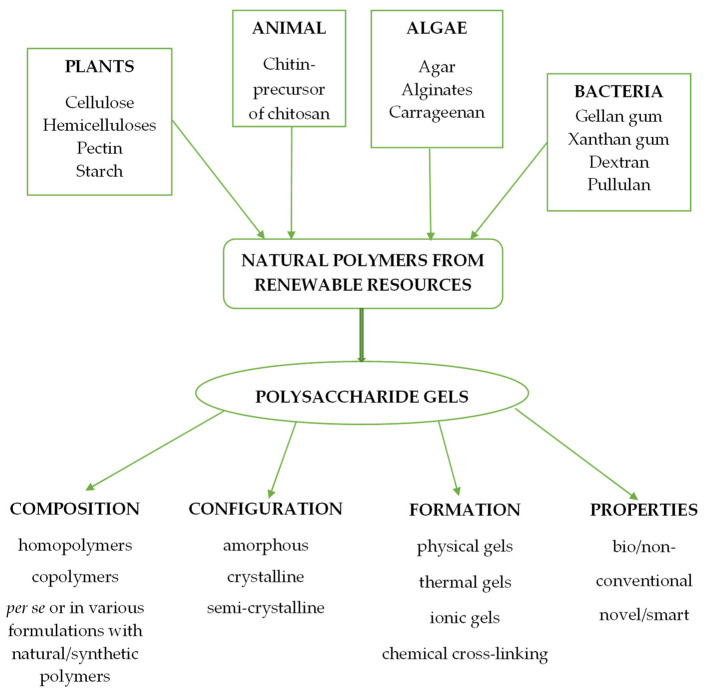
Polysaccharide gels: sources, types, and general properties.

Given their particular array of properties, gels prepared from colloid polysaccharides have been employed in a wide variety of applications [1] ranging from industry (food additives [2,3], packaging [4], pharmaceutics, and cosmetics [5,6,7,8], to healthcare and bioengineering (wound dressings [9,10,11], drug delivery systems [12,13,14,15], materials for tissue engineering [16,17,18,19], and prosthetics [20,21,22]. Their importance as a class of materials resides not only in their structural and functional properties, but in their multitude of structures, availability, and abundance in renewable resources as well (Scheme 1). The origin, processing, and further modification strategies influence the level of performance, and thorough investigations confirmed the possibility of tailoring the properties of these versatile materials.

Therefore, it was of high relevance to study their structure–properties relationship and the gelation mechanism under different conditions (temperature, pH). Furthermore, it was necessary to understand the principles that dictate all changes at molecular, macromolecular, and supramolecular levels. Comprehensive studies have focused on the gelation mechanism of polysaccharides [23,24,25,26,27,28,29,30,31,32,33,34,35] and emphasized that structural peculiarities are the result of the multilevel organization. It is well known that the primary structure refers to the sequence of monomer units in the macromolecular chains, the secondary structure indicates their spatial arrangement and orientation, while the tertiary structure is related to their supramolecular assembly as they pack in compact, stabile forms. For example, polysaccharides can adopt various helix and/or ribbon secondary structures in solution, and subsequently can develop complex supramolecular architectures (tertiary structure) as double helix or aggregates of helix and ribbons in gels [23]. 

All polymer systems present an entropic driving force to form random, disordered coils, so that favorable interactions are required to reach an ordered structure. In polysaccharides, these favorable interactions are mainly van der Waals attraction forces, hydrogen bonds, and dipole–dipole and ionic interactions. Thus, depending on the type of polysaccharide, some characteristic structures have been identified in polysaccharide gels: agarose and carrageenan series form double helix structures, while alginates and pectins achieve structures of ordered ribbons. 

Some basic rules of polysaccharide gelation have been substantiated [24,26] as follows: the gelation results from reaching stable tridimensional supramolecular structures by the formation of intra- and inter-molecular associations; pendant functional groups, such as hydroxyl (-OH), methyl (-CH_3_) or hemiacetal oxygen atoms, contribute to favorable interactions (van der Waals forces, hydrogen bonds), while other acidic groups in polysaccharides structure (sulfuric acid, carboxylic acid) form inter- and intra-molecular bridges by ionic bonds and electrostatic forces due to the presence of their corresponding cations.

Aside from these considerations, the significant role of water in the mechanism of polysaccharide gelation must be acknowledged. The molecule of water, H_2_O, can be involved in four hydrogen bonds: two by the hydrogen atoms, and two by the pair of lone electrons at oxygen atoms that bond other two hydrogen atoms from vicinal molecules. The resulting tetrahedral structure, which is the same as that of water in ice crystals, serves as a pattern for gelation of polysaccharide solutions, as demonstrated in the case of dilute amylose solution (1.0% *w*/*v*) when gelation occurred rapidly upon weak mechanical stimulation (cage effect) [36]. Another illustrative example is the formation of gel from agarose dilute solution [25]: agarose molecules are linked by hydrogen bonds inside the tetrahedral structures in water when a cooperative effect stabilized extended regions of structured water and agarose. Furthermore, oxygen atoms in monosaccharide units and pendant hydroxylic groups were involved in other hydrogen bonds, resulting in an extended cluster-like supramolecular association. Thus, gelation occurred rapidly at room temperature, when the agarose gel corresponds to the totality of stable clusters. The cage effect allows the lowest energy for lone electrons at oxygen atoms in hemiacetal groups and 3,6-anhydro-oxygen moieties. The tetrahedral conformation favors the hydrogen bonding in both distant water and agarose clusters and provides a hydrophobic effect as the outer surface abounds in carbon atoms. Recently, it was demonstrated that multi-stranded hydrogen bonds were formed in agarose molecules at high concentrations, which explained the successful employment of these gels in separation and purification processes applied to biomacromolecules such as DNA, RNA, proteins, and polysaccharides [37]. 

All these findings were supported by data obtained from different characterization methods (NMR spectroscopy, X-ray diffraction spectrometry, DSC, optical rotation, circular dichroism, and rheological investigations—bulk rheology and micro-rheology measurements, and computer modelling and optimization) that allowed scientists to confirm the polysaccharide gelation principles, as well as the structural peculiarities of the resulting materials.

**Scheme 2:**
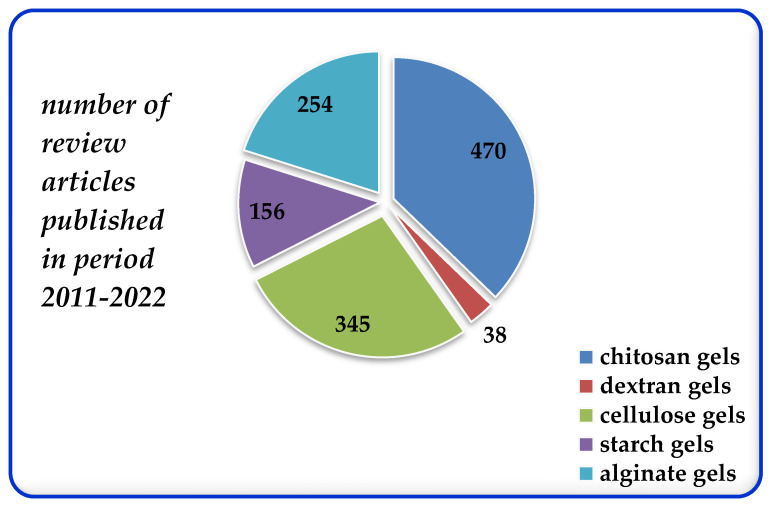
Volume of publications (reviews) on selected polysaccharide gels (source Web of Science).

In the present paper, some of the most widely used polysaccharide gels will be briefly reviewed with emphasis on their particular structure under various conditions. This approach has been conditioned by two factors. First, the topic—most of the published data focused on the practical/experimental side of the subject. Thus, new formulations and experimental protocols, and materials with improved and/or novel properties intended for different applications are constantly reported. Therefore, a significant number of review articles are published every year, as can be seen from the statistics published on the Web of Science for the last decade (Scheme 2), but only a few of them offer insights into structural issues. The present survey offers a complex approach of this topic, the structural peculiarities of polysaccharide gels, in correlation with the state of the polysaccharide (native or modified), formulation and processing parameters, the structure–properties relationship, and using modern methods or combined approaches of characterization. The second factor is that these few polysaccharides have been selected on the basis of their theoretic relevance, applicative potential, and volume of publications, criteria that designate them as being of high interest throughout the academic and industrial R&D community.

In recent years, a new approach emerged in the field of polysaccharide gels, namely combining different polysaccharides with each other or with other natural polymers (e.g., animal proteins such as gelatin and silk fibroin; vitamins; vegetal oils, etc.), synthetic polymers, micro-/nanoparticles and/or fibers, in order to obtain materials with improved or new properties. This enabled a larger number of formulations and materials to have special characteristics that make them fit for a more comprehensive range of applications. Some of the most relevant reports have been reviewed and presented as illustrative of this trend (Table 1).

## 2. Chitosan-Based Gel Materials

### 2.1. Chitosan-A Renewable Resource

Chitosan is a polysaccharide obtained by chitin deacetylation, the second most abundant after cellulose. In contrast to the raw material obtained from marine waste, which is a relatively inert polymer, chitosan is highly reactive due to the presence of the amino groups on its chains. This renewable resource is of animal origin, specifically from the exoskeleton of crustaceans such as crab, lobster, shrimp, etc. Chitin extraction from marine waste comprises three main steps: (i) *decalcification* in HCl diluted aqueous solution, (ii) *deproteinization* by treatment with NaOH diluted aqueous solution, and (iii) *depigmentation* with KMnO_4_ and oxalic acid solution. As previously stated, the resulting polysaccharide is relatively inert from a chemical point of view, being soluble in aqueous alkaline solutions [50]. 

To obtain chitosan, chitin is subjected to a deacetylation reaction by treatment with concentrated NaOH solutions of 40–50%. This aggressive pH renders not only the cleavage of the amide bond, but it also leads to scissions of the polysaccharide macromolecular chain, yielding products with different molecular weights (MW) and a high polydispersity index. It is usually accepted that the polysaccharide with a deacetylation degree (DD) higher than 50% is considered chitosan, since it exhibits specific behavior, such as solubility in diluted acidic aqueous solutions [51]. However, commercially available chitosan customarily has a DD of over 75%; for higher DD of over 95%, the MW is heavily impacted and only oligomers and small saccharide structures are obtained. In addition, depending on the subsequent material processing, chitosan with either high, medium, or low MW can be chosen. For example, particle preparation requires a polysaccharide with shorter chains, compared to a bulk hydrogel, where even high MW chitosan can be suitable. Additionally, solution properties, such as viscosity, change drastically with the variation of DD and MW of chitosan.

### 2.2. Properties—Influence of the Structure

**Scheme 3:**
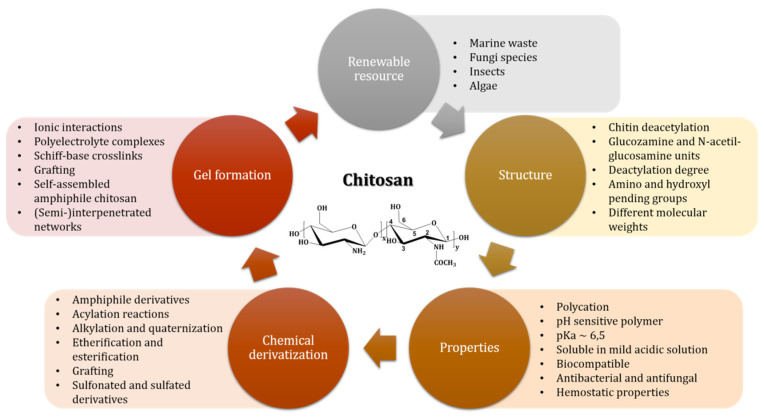
Chitosan—from origins to complex formulations for specific applications.

Chitosan is perhaps the only polysaccharide with amino moieties in its structure. This entails unique properties and gives grounds for the massive interest of researchers in using this macromolecule; particles, hydrogels, films, membranes, and even composites and coatings have been reported for any number of applications, e.g., wound dressing, tissue engineering, drug delivery, cell encapsulation, bioimaging, sensors, textiles, wastewater purification, fertilizer reservoir, etc. [52]. Scheme 3 is illustrative for the complexity of this field of research. 

It is of paramount importance to underline that the high number of amino groups present on the polysaccharide chain makes chitosan a polycation. Depending on the solution pH, the amino groups are either neutral or as ammonium ions. Usually, chitosan has a pKa of around 6.5, but it depends on the degree of N-deacetylation. Therefore, at acidic pH chitosan is soluble, while at alkaline pH it precipitates. This behavior was used as the main mechanism to prepare hydrogel beads, and the reported materials prove to be stable over a long period of time [14]. Moreover, the materials’ response in terms of swelling degree variation in accordance with environmental pH was observed and implemented for DD and sensing applications.

Chitosan is not only a pH-sensitive polymer, but it maintains this behavior when included in polymer blends or crosslinked 3D networks [53,54]. Furthermore, the amino groups enable responsiveness to ionic strength and various metal ions, feature which is highly favorable for wastewater purification materials [55].

From a biomedical point of view, chitosan is a biodegradable and biocompatible polymer with antibacterial and antifungal activity [56]. If the first two are due to the polysaccharide nature of chitosan, the last two properties are a consequence of the particular structure.

Chitosan is naturally degraded by the chitosanase enzyme, but studies have proved that for biomedical applications chitosan is biodegradable in the presence of lysozyme, an enzyme found in most mucous tissues [56]. Specifically, it has a minimal number of six consecutive amino-glucose units to attach to in order to cleave one glycosidic bond [57,58]. When assessing the chitosan-based materials’ stability under in vitro conditions in the presence of lysozyme, one must take into consideration the polysaccharides MW, since lower MW entail faster degradation rates.

The biocompatibility of chitosan is an aspect upon which not all researchers share a unanimous opinion. Even the definition of biocompatibility refers to the fact that the results induced by a biomaterial must be correlated to a specific function and application. However, good and bad cytotoxicity data was reported in the literature, depending on factors such as polymer or particle concentration, type of material, type of cells, purity degree of crosslinked scaffolds, electrical charge, method, etc.

Much attention was given to antibacterial and antifungal properties of chitosan-based materials. In-depth studies were reported, aiming to shed light on the mechanism involved in this phenomenon and multiple hypotheses were investigated [59,60]. It was found that the most probable mechanism behind the antibacterial activity of chitosan lies in the interactions between the amino groups of the polysaccharide with the negatively charged glycosaminoglycans that are present on the surface of the bacterial wall. The polyelectrolyte complexes induce the disruption of the membrane and the extravagation of the content, which means the apoptosis of the bacterium cell.

Another important property is the hemostatic action of chitosan. This feature was readily applied in wound dressing for combat hemorrhages, as well as in the clinic for the management of burns and traumatic injuries. Again, the amino groups are responsible for the rapid arrest of the bleeding as a consequence of their interaction with the membranes of the red blood cells that bear negative charge; thus, the aglutination of platelets and red blood cells has been initiated, which entailed activation of thrombin. As a result, the clotting pathway is activated, and the thrombus formation occurred [61].

### 2.3. Chemical Functionalization of Chitosan

Despite the numerous advantages brought about by the presence of the amino groups in the structure of chitosan, this polysaccharide remains difficult to process in aqueous solutions on one hand, and, on the other hand, this highly reactive moiety provides a great opportunity for chemical modifications.

A plethora of reactions were explored and various reviews have been published on this topic [62,63,64]. Some of these reactions aim at improving the solubility in water, while others target a specific type of material. For example, amphiphile chitosan was synthesized for self-assembling particles either polymeric or composite with magnetite for medical applications [65]. Another type of reaction is grafting chitosan with polymerizable groups such as maleoil or citraconil [66,67]. The C=C bonds can be successfully subjected to crosslinking using acrylic acid or its derivatives, either alone or as copolymers.

In contrast to the multitude of possibilities concerning the chemical modification of chitosan at any of its three functional groups on each glucosamine unit (two -OH and one -NH_2_ groups), some reactions are not possible due to the high reactivity of the amino groups. For example, selective oxidation reactions were attempted for the introduction of aldehyde or carboxyl groups using well-known protocols, i.e., TEMPO-oxidation or periodate-oxidation [68,69]. With few exceptions given by special reaction conditions, all authors reported the aggressive degradation/intense depolymerization of chitosan and no applicability of the reaction given the extremely low yields. 

### 2.4. Chitosan-Based Gels

Given the presence of the amino groups on the polysaccharide backbone of chitosan, there is a plethora of possible gelling mechanism. Hydrogels can be prepared by either physical crosslinks, chemical crosslinks, or both. Despite this rather simplistic, generally accepted classification, the existent possibilities for the hydrogel network formation are indeed extremely vast. Several examples will be discussed in the following paragraphs.

Ionic interactions can be successfully utilized as previously described. Compared to the case of alginate or other COOH bearing polysaccharides, where metallic di- or polyvalent ions can be added to form intercatenar bonds, chitosan interacts differently. Stable beads and particles were obtained by changes in pH [14], leading to rearrangements in chitosan conformation and self-crosslinking. Another method is the addition of sodium tripolyphosphate to a chitosan solution. Such networks form homogenous porous hydrogels that are relatively stable under physiological conditions [70].

Polyelectrolyte complexes are also a good opportunity. Many polymers with COOH groups, either natural (alginate, hyaluronate) [71], naturally derived (TEMPO oxidized polysaccharides, e.g., cellulose, pullulan) [14], or synthetic (polyacrylic acid, polymethacrylic acid) [11,17,67,72], were explored to prepare chitosan-based gels.

Self-healing hydrogels based on Schiff base linkages attracted a lot of attention. They are easily obtained using chitosan as one partner and an aldehyde-bearing compound as another. On these types of materials, authors have reported on hydrogels with various types of compositions. Due to the ease of preparation and high yield, periodate-oxidized polysaccharides are an excellent choice to associate with chitosan. Dialdehyde cellulose, pullulan, alginate, pectin, and hyaluronan were studied in different ratios and with different oxidation degrees [73,74]. It was observed that an increase in aldehyde concentration positively impacts the mechanical resistance and the self-healing capacity, but negatively affects the gelation time, swelling behavior, and biocompatibility. Therefore, due the great advantages of self-healing, injectability, fine-tuning and biocompatibility, these hydrogels have real potential in medical application. Further studies are needed in the area of gels for cell encapsulation aiming to restore tissue integrity, but drug loaded materials and tissue fillers are already in clinical use. Generally, chitosan is easily characterized by means of FTIR and NMR spectroscopy. However, some of the structural changes in chitosan derivatives and some types of crosslinks are less evident in these types of spectra. For example, the presence of Schiff base bonds could be confirmed by high resolution XPS spectroscopy [14], since in the FTIR and NMR spectra the imine bond gives low intensity signals and overlaps other bands or peaks [75].

Chitosan functionalized with long alkyl chains represents a class of derivatives with peculiar behavior as compared to the raw material or other types of chitosan-derived products. The substitution may occur at either hydroxyl or amino moieties, or both, depending on the reagent and reaction conditions [63,74]. Their capacity to self-assemble gives opportunity for the preparation of micelles, liposomes, or nanocomposites, but it can also lead to the formation of hydrogels [76]. Interestingly, these hydrogels also have the capacity to be injectable and have self-healing behavior due to their gelling mechanism, i.e., supramolecular assemblies in hydrophile/hydrophobe domains, but since they are not stable under physiological conditions, they did not find much applicability as gels at the macroscale [77].

Chemical crosslinking by grafting and synthesis of semi-interpenetrated networks are two methods of preparing chitosan-based hydrogels by association with synthetic polymers. Chitosan can be successfully crosslinked with acrylates by homo- or co-polymerization [11,78,79]. The resulting materials exhibit pH-sensitivity and an excellent swelling capacity. Their properties can be easily tuned, given the nature of such reactions: chitosan/monomer ratio, comonomers ratio, initiator concentration, etc. Moreover, they prove to be suitable for a variety of applications, ranging from cartilage substitute to reusable materials for wastewater purification.

## 3. Dextran-Based Gels

Dextran is a bacterial polysaccharide composed mainly of α-D-glucopyranose units connected to each other by α-1,6 glycosidic bonds, with a lower branching degree via α-1,3-connected side chains. This polysaccharide is broadly used for biomedical applications (blood-plasma substitute, peripheral flow enhancer, antitrombolytic agent, artificial tears, etc.) due to its biocompatibility, hydrophilicity, and lack of toxicity. The high density of its hydroxyl groups afforded the obtaining of soluble or crosslinked macromolecular structures. Dextran-based hydrogels have been depicted in the literature either chemically or physically crosslinked. Chemically dextran gels can be achieved by radical polymerization or chemical reaction of functional groups having complementary reactivity. Physically crosslinked dextran hydrogels were acquired by ionic interaction, crystallization, or stereocomplex formation. The main uses of dextran-based gels were for biomedical purposes (delivery of proteins, drugs, or enzymes, hypolipidemic effect, tissue adhesives, tissue engineering, antibacterial and antifungal matrices, and wound dressings), wastewater purification, and so on. 

### 3.1. Chemically Crosslinked Dextran Gels Obtained by Radical Polymerization 

Acryldextran, acquired by the reaction between dextran and glycidylacrylate, was polymerized in the presence of N,N,N′N′-tetramethylene-diamine and ammonium peroxydisulfate as initiator system [80,81,82]. The obtained polymer was used for the immobilisation of enzymes and drugs. Enzymes, incorporated in polymeric microspheres by an emulsion polymerization method, showed a complete retaining of their activity [81,82]. A quantitative functionalization of dextran was carried out using glycidylmethacrylate by using 4-(N,N-dimethylamino)pyridine as the catalyst. Mass spectroscopy and nuclear magnetic resonance spectroscopy, employed for the characterization of the obtained polymer, revealed that the methacrylate group was successfully bound to the dextran backbone [83]. Enzyme kinetics, gel permeation chromatography, and electrospray mass spectrometry showed that long unsubstituted chains (18 or more glucopyranosic units) of dextran gels were enzymatically hydrolyzed at the same rate and level as pristine polysaccharide, while unsubstituted chain segments (6 to about 18 glucopyranosic residues) were hydrolyzed slower. Shorter unsubstituted chain segments were not enzymatically degraded [84,85,86,87]. The reaction between dextran and maleic anhydride a monomer with vinyl groups to be obtained. Its UV-induced polymerization a hydrogel to be obtained which was not degradable under physiological conditions. Polymeric swelling ratio showed a strong pH and degree of substitution dependency due to the generation of ionizable carboxylic acid groups by the reaction of dextran with maleic anhydride [88].

Derivatives of methacrylate dextran, having the polymerizable groups connected to dextran skeleton via a carbonate ester, were prepared by radical polymerization [89,90]. Gels obtained after the polymerization could degrade (by chemical hydrolysis) under physiological conditions due to the occurrence of hydrolytically sensitive groups in the crosslinks. Gels’ degradation degree and time can be modified by the length of the spacer in polymeric crosslinks. These biocompatible polymers, achieved as injectable microspheres, were appropriate for the controlled delivery of recombinant human interleukin-2 [90,91]. Microspheres, synthesized by using poly(ethylene glycol) and methacrylated dextran, could entrap and release protein model (liposomes) in the integral form and size, in a pulsed and sustained way. Encapsulation in microspheres stabilizes liposomes and can help absorption via M-cells [92]. Dextran, having acryloyl chloride [93] or allyl isocyanate [94] (hydrophilic constituent) as polymerizable groups, reacted with poly(D,L-lactic acid) diacrylate (hydrophobic constituent). The resulting linear polymers were subjected to UV radiation allowing the formation of a polymeric network which was used for the release of bovine serum albumin (BSA) [93]. Release of protein was conducted by diffusion of BSA via swelling of the hydrophilic phase during an initial stage, and degradation of the hydrophobic phase during a delayed stage. The extent of diffusion against degradation-controlled release depended on composition ratio and dipping time [95]. An efficient hydrogel, used in dyes’ adsorption, was synthesized by utilizing glycidyl methacrylate modified dextran and acrylic acid. The gel showed high elimination efficacy on Methylene Blue and Crystal Violet for a broad pH range (3–10) as a result of the polymeric network’s strong buffer capacity [96]. Removal efficiencies for dyes were still higher than 95% after five adsorption/desorption cycles. 

### 3.2. Chemically Crosslinked Dextran Gels Achieved by Reaction of Complementary Groups

Crosslinked polymers could be also obtained by the chemical reaction between their functional groups with complementary reactivity. Thus, gelatin was crosslinked with polyaldehydes acquired by partial oxidation of dextran [97]. These gels, having epidermal growth factor included in their matrix, were projected for wound treatment. A prolonged storage of the epidermal growth factor determined the decrease of delivery rate due to the obtaining of Schiff bases between gelatin ε-lysine groups and oxidized dextran aldehyde groups. The biocompatibility of such gels was studied in vitro and in vivo and was esteemed as satisfactory [98].

**Figure 1:**
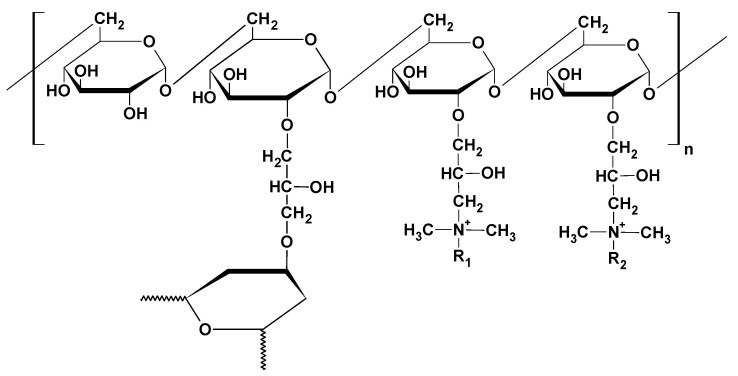
Dextran-based hydrogels (R_1_ = dodecyl or hexadecyl; R_2_ = ethyl).

Crosslinked dextrans were synthesized in alkaline medium (NaOH) by use of different monomers containing Cl-, P- and N (epichlorohydrin, phosphorus oxychloride and N,N’-methylenebisacrylamide) [99]. Polymeric microspheres based on this polysaccharide were synthesized in a water-in-oil dispersion, with epichlorohydrin as crosslinking agent and cellulose acetobutyrate as stabilizer [100]. Further, crosslinked dextran could be functionalized with different compounds for obtaining polymeric networks with various applications. So, amphiphilic cationic dextrans, carrying one type of quaternary ammonium groups as pendant groups, were achieved in aqueous solution by one pot procedure, implying the reaction between the polysaccharide and a equimolar mixture containing epichlorohydrin and a tertiary amine (dimethylalkylamine, where alkyl = C_2_, C_4_, C_8_, C_12_, C_16_, etc.) [100]. Gels, having two types of side-chains with N,N-dimethyl-N-alkyl-N-(2-hydroxypropyl) ammonium chloride groups containing different alkyl chain length substituents (C_2_ and C_12_/C_16_, respectively) (Figure 1), were achieved by two successive quaternization reactions of crosslinked dextran with the equimolar mixture of epichlorohydrin/tertiary amine. 

**Figure 2:**
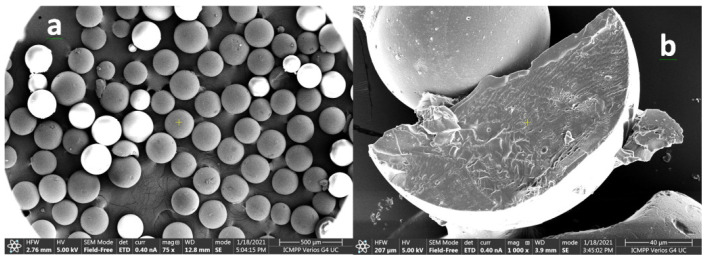
SEM images of dextran microparticles, magnified 74 times (**a**), microparticles in cross-section, magnified 1000 times (**b**).

Dextran-based amphiphilic gels with pendant quaternary ammonium groups were studied as potential anticholesteremic agents or efficient dye adsorbents. The occurrence of two types of pendant quaternary ammonium groups in gel structure enhanced the above-mentioned properties. [101,102,103,104]. The morphology of dextran cationic hydrogels was analyzed by scanning electron microscopy. The polymeric microparticles, observed in dry state, were perfectly spherical, having a diameter between 100–220 μm (Figure 2a). These hydrogels are not porous in dry state (Figure 2a,b), but they have a high water uptake capacity (swelling porosity) which favors the diffusion of adsorbate molecules inside the swollen network of the microspheres.

Adsorption studies, performed in water and 10 mM NaCl solution, by using dextran gels (Figure 1) as sorbents and sodium bile salts (sodium cholate and sodium deoxycholate) as adsorbates [101,102]. Factors that influenced the sorption process were lipophilicity and water retention of the gel, and adsorbate hydrophobicity and ionic strength of the medium. All amphiphilic polymers showed a better affinity and capacity for binding in comparison with commercial Cholestyramine^®^. The same charged hydrophobically modified polymers, containing two types of side-chains groups with different polarities (hydrophob/hydrophyl), were used for dye sorption studies [103,104]. Thus, adsorption capacities for Methyl Orange, Orange II, Indigo Carmine, and Rose Bengal were evaluated as a function of hydrogel characteristics by using equilibrium, kinetic, and thermodynamic analysis. The influence of contact time, initial dye concentration, dye type, pH and temperature on adsorption process were also studied. The sorption capacity of the above-mentioned hydrogels was greater, for the same dyes, in comparison with hybrids like inorganic-polymer. A successive addition of water, NaCl 0.5 M, and methanol afforded the complete regeneration of the hydrogel.

### 3.3. Physically Crosslinked Gels Acquired by Ionic Interaction

Often the crosslinkers are harmful products that could change the safety of the active compounds entrapped in the polymeric matrix. Various techniques have been used for the formation of physically crosslinked polymers. One of these methods is the crosslinking using the addition of different ions. This process does not need the occurrence of charged functional groups in the polymeric structure. So, dextran, which is a neutral polysaccharide, forms a gel in the presence of potassium ions due to the formation of a cage, with the help of the oxygen atoms of α-D-glucopyranosidic units of the polysaccharide, in which the metal ions completely matched. These complexes were unstable in water and were not appropriate for drug delivery objectives [105].

### 3.4. Physically Crosslinking Gels Obtained by Crystallization

Dextran 6000 freely could form a hydrogel by stirring its concentrated aqueous solutions at room temperature [106]. The association of dextran chains via hydrogen bonding determined a crystallization process which afforded the achievement of polysaccharide-based microspheres. The concentration of the polymer solution influenced hydrogel features. 

### 3.5. Physically Crosslinking Gels Acquired by Stereocomplex Formation

Dex-(L)lactate and dex-(D)lactate were obtained by the coupling reaction between native dextran and terminal hydroxyl group of L- and D-lactic acid oligomers, respectively [107]. A new polymer was achieved at room temperature by mixing the aqueous solutions of resulted polymers. Rheological measurements proved dextran gel formation by the determination of its storage modulus variation at different temperatures. So, storage modulus decreased with the increase temperature up to 80 °C and returned to initial value by cooling up to 20 °C, showing the thermodynamic reversibility and the physical nature of crosslinking. Dextran-based stereocomplex hydrogels loaded with model proteins (IgG and lysozyme) were obtained by mixing the solutions of protein and dextran-g-oligolactate [108]. Hydrogels are completely degradable under physiological conditions. The chemical composition of the polymer influenced the time required for its degradation (1 to 7 days). The release of both model proteins from the gel was qualitative and the enzymatic activity of one of the model proteins (lysozyme) was fully preserved.

## 4. Starch-Based Gels

Starch is the main component in wheat, contributing to the characteristics of wheat-based foods that include moisture retention, viscosity, texture, taste, and shelf-life [109]. The quality attributes of starch-containing products result from the specific gelatinization and retrogradation behavior of starch. Gelatinization of starch occurs over the critical temperature in the presence of sufficient water; it is an irreversible phase transition process initiated by hydration and swelling of the amorphous region of starch [110]. 

**Scheme 4:**
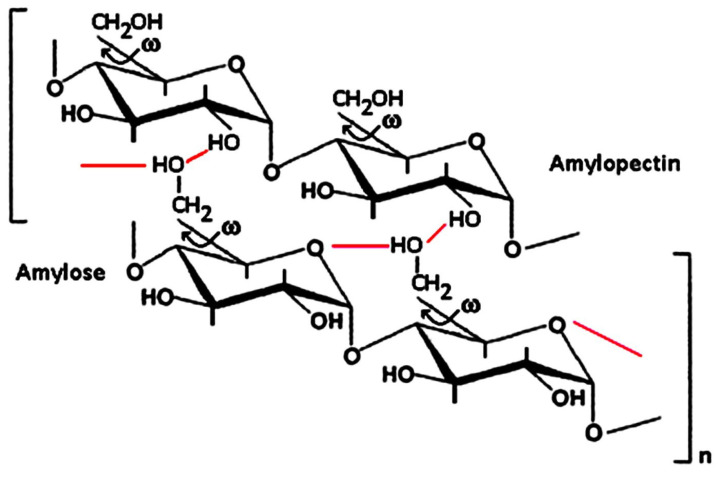
Gelatinization mechanism of starch. The red lines represent hydrogen bonding.

Generally, starch granules show their intact shapes before gelatinization as observed under normal light, are birefringent and show the characteristic “Maltese cross” pattern under polarizing light. Birefringence patterns indicate a radial alignment of crystallites within starch granules, and the loss of birefringence on heating, which is indicative of disordering processes, suggests the loss of radial-aligned crystallites [111]. When the loss of birefringence begins, most of the granules are partially swollen. The regular orientation of D-glucosyl units in amorphous and crystalline regions disappears, the swelling becomes irreversible, and the characteristic “Maltese cross” pattern ceases to be visible under polarizing light with increase in heating temperature. Finally, the birefringence of starch granules vanishes completely; the disrupted parts of the granules become the same color as the background, and the gelatinization process is over. During starch gelatinization, the disruption of crystallinity in a particular area of the granule increases the swelling capacity of this area. The swollen disrupted parts of the granule have much higher water content than the amorphous parts of the undisturbed area. Obviously, the swelling of disturbed areas accelerates the process of disruption of neighboring crystalline regions, and that this process rapidly propagates along the granule [112]. A gelatinization mechanism of rice, potato, and wheat starch might take place by the intermolecular association between the O-6 of amylose and the OH-2 of amylopectin molecules through hydrogen bonds, as illustrated in Scheme 4 [26]. 

The gelatinization and retrogradation of starch are affected by various endogenous and exogenous factors including amylose to amylopectin ratio, structures of amylose and amylopectin, the packing of amylose and amylopectin chains in the granules, the amounts of minor endogenous components (e.g., protein, phosphorus in various forms, and lipids), moisture content of the system, and experimental conditions (e.g., pressure, heating and cooling speeds, and history) [26,113].

Starch gelatinization and associated properties can be evaluated by various methods, including optical microscopy, electron microscopy, differential scanning calorimetry (DSC), X-ray diffraction (XRD), nuclear magnetic resonance spectroscopy NMR), Fourier transform infrared spectroscopy (FTIR), atomic force microscopy, viscosity measurement, enzymatic digestibility, light extinction, and solubility or sedimentation of swollen granule [114]. All these methods measure slightly different physicochemical properties and have unique and inherent advantages and disadvantages. 

It has been reported that amylopectin has short branch chains that can form a double helix crystalline, and the double helix crystalline would be torn apart during gelatinization [115]. These short-branched chains retain a certain “memory” in a regular pattern after gelatinization [116]. They tend to form gel-balls mainly composed of chains from the same sub-main chain. Moreover, an amylopectin molecule might form a relatively separate large cluster and super-globe. The molecular entanglement between the gel-balls and super-globe is less than that between linear polymer chains due to their smaller size and short length of these amylopectin chains. Consequently, these gel-balls formed by amylopectin require less energy to move than those formed by long linear chains.

Starch gelatinization is an endothermic transition process, in which the ordered single and double helices are dissociated to an amorphous conformation [117]. Therefore, the variations in thermal properties can reflect the changes of helical structure within starch granules to some extent. The gelatinization temperature range (Tc-To) reflects the degree of homogeneity of starch crystallites [118]. The onset temperature (To) is the melting temperature of the weakest crystallite in starch granules, while the conclusion temperature (Tc) represents the melting temperature of high-perfection crystallite [119]. The enthalpy of gelatinization (ΔH) mainly reflects the loss of double helices, which is related to the content and stability of double helices [120]. The results of DSC concerning starch gelatinization temperature and enthalpy are associated with thermal melting of crystallites/helical structures, while XRD is used to quantify crystallinity of crystallites structures, and ^13^C CP/MAS NMR can further quantitatively investigate the helical structure. The higher content of double helices corresponds to the higher crystallinity, while the content of double helices always exceeds the value of crystallinity. The result indicates that not all double helices are within crystalline arrays [121]. In addition, the higher content of double helices and crystallinity matches with the higher gelatinization temperature and enthalpy in the same kind of starch, while this phenomenon varies slightly in different kinds of starch, because starch thermal properties are also related to the stability of starch crystalline or helical structure [122].

The gelatinization processes of twelve starches were in situ analyzed using a polarizing microscope in combination with a hot stage, and four patterns of crystallinity disruption during heating were proposed. The crystallinity disruption initially occurred on the proximal surface of the eccentric hilum, on the distal surface of the eccentric hilum, from the central hilum, or on the surface of the central hilum starch granule. The patterns of initial disruption on the distal surface of the eccentric hilum and on the surface of the central hilum starch were reported for the first time. The heterogeneous distribution of amylose in starch granule might partly explain the different patterns of crystallinity disruption and swelling during gelatinization [123]. 

The gelatinization of waxy (very low amylose) and high-amylose maize starches by ultra-high hydrostatic pressure (up to 6 GPa) was investigated in situ using synchrotron XRD on samples held in a diamond anvil cell (DAC). The starch pastes, made by mixing starch and water in a 1:1 ratio, were pressurized and measured at room temperature. X-ray diffraction pattern showed that 2.7 GPa waxy starch, which displayed A-type XRD pattern at atmospheric pressure, exhibited a faint B-type-like pattern [124]. When exposed to very high pressure (>2.5 GPa), both waxy and high-amylose maize starches could be fully gelatinized, and XRD diffractograms showed total loss of crystallinity. When the pressure was released back to atmospheric pressure, waxy maize starches showed broad XRD peaks likely due to amylopectin reaggregation, an effect known to be at the origin of starch retrogradation. It is important to stress that this study was carried out at a starch to water ratio of 1:1, and it is expected that the effect of high hydrostatic pressure HHP on starch crystallinity will depend on this water content. Overall, this study demonstrates the potential of using synchrotron XRD in combination with a DAC to monitor in situ starch gelatinization at pressures exceeding the ambient pressure.

Retrogradation is a recrystallization process in which disaggregated amylose and amylopectin molecules in gelatinized starches reassociate to form ordered structures. Starch retrogradation takes place in two stages. The first phase of retrogradation (short-term retrogradation) occurs as the network formed between amylose molecules as paste cools down, forming a fresh elastic gel. Amylose retrogradation determines the initial hardness of a starch gel, and the stickiness and digestibility of processed foods. The second phase of retrogradation (long-term retrogradation) is associated with recrystallization of the outer branches of amylopectin [125]. The long-term retrogradation behavior of starch affects the long-term development of gel structure and crystallinity of processed starch [126]. Controlling retrogradation behavior and gel properties of starch has been crucial to control the quality of starch-based products [127]. 

**Scheme 5:**
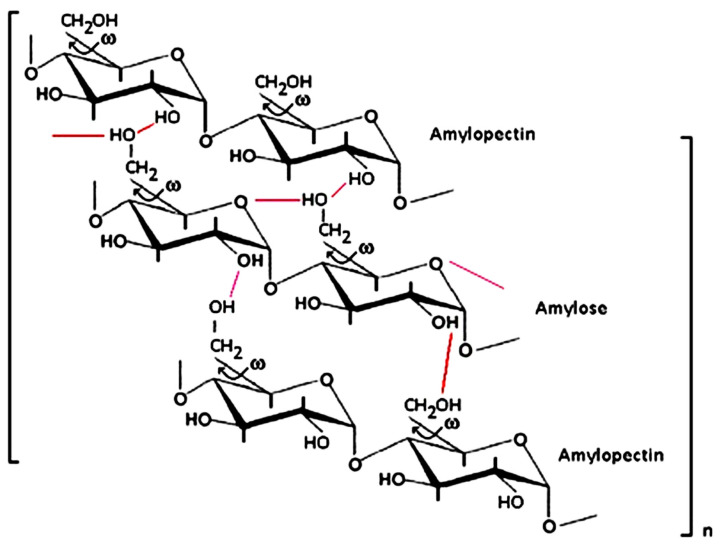
Retrogradation mechanism of rice, potato, and wheat starch. The red lines represent hydrogen bonding.

During retrogradation, intermolecular hydrogen bonds might form between the OH-2 of a D-glucopyranosyl residue of the amylose and the O-6 of a D-glucopyranosyl residue of short side-chain of the amylopectin molecules, as illustrated in Scheme 5 [26]. In addition, an intermolecular association through hydrogen bonds occurs between amylopectin molecules due to a decrease in Brownian motion and kinetic energy of amylopectin and water molecules during storage. The side-by-side associations between the O-3 and the OH-3 of D-glucopyranosyl residues on different amylopectin molecules may also take place.

The native structure of starch under various food processing conditions exhibits low resistance to high shear rate, thermal decomposition, high retrogradation, and syneresis. These shortcomings can be overcome by modifying the native structure of starch by chemical, physical, or enzymatic methods to obtain the desired physico-chemical properties such as structure, adhesion, texture, heat tolerance, water solubility, gelatinization, retrogradation, pasting, viscosity, swelling, and syneresis parameters [128].

Heat moisture treatment is carried out by heating of starch granules at an elevated temperature of around 110 °C (well above gelatinization temperature) but with limited moisture levels generally varying from 20 to 30% for a certain period of (up to 16 h). Heat moisture treatments change pasting properties and decrease the swelling power and solubility of HMT starch rendering the starch more resistant to heat, acids, and mechanical deformations [129]. 

Heat-moisture treatment (HMT) at different moisture content applied on breadfruit starch evidenced an increase in the gelatinization temperature range (Tc–To), and a decrease in gelatinization enthalpy (ΔH) in comparison with those for the native starch. The difference in gelatinization temperature may be attributed to the difference in amylose content, size, form, and distribution of starch granules, and to the internal interaction and/or realignment of starch chains within the granule. The melting temperatures (To, Tp, and Tc) of starch crystallites are controlled indirectly by the surrounding amorphous region [130]. After HMT, the starch became more closely packed in crystalline and non-crystalline structures, resulting in reduced granule swelling, which would reduce the destabilization effect of the amorphous region on the crystallite melting. Therefore, a higher temperature would be required to melt the crystallites of HMT-modified starches. To, Tp, and Tc were increased with higher moisture content. The higher (Tc–To) values after HMT might be caused by the rearrangement of molecules forming some ordered and stable structure. Additionally, HMT led to starch with greater thermal stability as the transition was moved to higher temperatures [131]. The transition temperatures To and Tc are related to the melting of the weakest and strongest molecular structures, respectively, while the gelatinization enthalpy (ΔH) represents the amount of energy required for disrupting these structures [132]. In other words, To and Tc represent the perfection of the ordered structures, and ΔH reflects the content of them.

The effect of moisture level (20%, 25%, and 30%) and heating length (2, 4, 8, and 16 h) on the physicochemical and structural properties of normal maize starch and waxy maize starch during HMT indicated that both moisture content and heating length affected the properties of maize starches to a large extent [133]. The study evidenced that, in addition to being more highly altered than amylopectin after chemical modification of starch, amylose played an essential role in physical modifications of starch by annealing and HMT. The rearrangement of amylose was associated with the presence of water molecules, which might serve as a plasticizer, leading to higher rigidity of starch granular structure in amorphous regions that might disrupt the organized crystalline structure of the starch granule. HMT can influence the structure and physicochemical properties of cereal, tuber, and legume starches, as reflected by significant changes in the X-ray diffraction (XRD) pattern, crystallinity, granule swelling, amylose leaching, gelatinization parameters, viscosity, thermal stability, rheological characteristics, and acid/enzyme susceptibility [134,135,136,137]. While the HMT-induced changes to the starch structure and properties have been found to depend on the starch source and treatment conditions (e.g., temperature, moisture, and time), generally HMT starches tended to have a higher gelatinization temperature, lower paste viscosity, a decrease in the granule swelling, and an increase in the thermal stability [138,139]. HMT decreases starch solubility, swelling power, amylose leaching, and peak viscosity, and increases the pasting temperature [140,141]. A study regarding the relationship between the degree of starch gelatinization (DSG) and its physicochemical and structural properties involved potato starch samples with DSG ranging from 39.41% to 90.56% obtained by hydrothermal treatment. The endothermic enthalpy, gelatinization range, and short-range ordered structure of starch were negatively correlated with DSG, while onset gelatinization temperature, apparent viscosity, and water-binding capacity were positively correlated. Starch granules gradually lost their typical shape, and less birefringence was observed with increasing DSG [142]. The extent of the changes in these physicochemical characteristics is mainly influenced by the alteration of the semi-crystalline supramolecular structure in starch granules [143]. Generally, HMT results in structural changes within both amorphous region and crystalline region to different degrees. The greatest changes were proposed to occur in the amorphous region through facilitating the chain associations in cereal, tuber, and legume starches [144]. It is difficult to define the properties of HMT starches in a consistent way because the reaction conditions are diverse, including the botanical source, moisture content, temperature, length of treatment, heat resource, and cooling process. 

Annealing can be defined as a process which involves incubation of starch granules at a temperature above the glass transition (T_g_) but below the onset of gelatinization (T_o_) temperature, usually at or above 40% water *w*/*w* [139]. Annealing causes a reorganization of starch polysaccharide associations which modify the physical properties without destroying the granule morphology. This physical transformation is associated with decreased swelling power and solubility [145], an increase in gelatinization temperatures and enthalpy, plus a narrowing of the gelatinization range, increased paste stability and crystallinity with a decrease in peak viscosity and rate/extent of retrogradation [146]. 

Annealing treatment could significantly increase the onset temperature (To), gelatinization enthalpy (ΔH), particle size, peak viscosity, breakdown, final viscosity, and setback values of early *indica* rice starch, and could significantly decrease the gelatinization temperature range (Tc–To) and tan δ (loss factor represents the ratio of viscosity to elasticity of the gels) value of the starch [147]. Annealed and HMT water chestnut starches exhibited marked changes in their physicochemical, gelatinization and pasting properties, namely higher gelatinization temperature with reduced gelatinization temperature range but increased gelatinization enthalpies [148]. 

Chemical modification involves the introduction of new functional groups into the starch molecule which produces significant improvements in starch physicochemical properties such as solubility, gelatinization, pasting, and retrogradation. The functional properties of the modified product depend on the nature of substituents (including acetyl, hydroxypropyl, phosphate (monoester), carboxymethyl, octenyl succinic, and cationic groups), degree of substitution and distribution of substituents in the starch molecule, and also on the reaction conditions such as nature (like phosphoryl-chloride, sodium tripolyphosphate, vinylchloride, mono-sodium phosphate, and epichlorohydrin) and concentration of reagents, reaction time, type of catalyst, pH, and temperature [149].

The chemical modification with acetylation, hydroxypropylation, and cross-linking significantly affected the physicochemical properties of rice starches. Dual-modification, acetylation and hydroxypropylation of cross-linked rice starches, provided desirable changes in the functional properties with increased gelatinization temperature, gel hardness, and gel chewiness and decreased breakdown, swelling, and solubility, which could enable a wide range of applications. The extent of change in the functional properties after the dual modification of *japonica* and *indica* rice starches was different, possibly due to the differences in the amylose content, molecular structure of amylopectin, granular swelling, degree of substitution, and location of substituent groups [150]. 

Blending of native starches from different sources is a common physical method for tuning the textural properties of the final product. This method could enable the use of starch in the industrial processing without modifying the structure of the native starches. It has been reported that the blends of high amylose corn starch and waxy cassava starch produced higher hardness of starch gels [151]. Dry heating of maize starch–hydrocolloid mixtures under different pH conditions induced changes in the textural properties of the stored starch gels, such as low hardness, springiness, cohesiveness, and chewiness of stored starch gels, were observed by incorporating xanthan and sodium alginate during dry heating [152]. 

Starch and non-starch polysaccharide complexes have been used in processed foods to overcome the shortcomings of native starches, for example, to protect starch granules against shear, improve product texture/rheology, and hold moisture. The effects of various non-starch polysaccharides with different molecular characteristics and additive amounts on starch-containing gels, such as xanthan gum, guar gum, gellan, or carrageenan, have been studied extensively. Soluble soybean polysaccharide had different anti-retrogradation effects on different types of starches [153,154]. The gel properties of starch gels, such as viscoelastic properties and textural properties, have been investigated in starch–hydrocolloids mixed gels. The storage modulus and hardness of *Mesona chinensis* polysaccharide–starch mixed gels increased with the increasing of polysaccharide concentration [155]. The network structure formed between starch and tamarind seed polysaccharide molecules enhanced the elastic properties of starch-containing gels [156].

Gelatinization, retrogradation, and gel properties of wheat starch–wheat bran arabinoxylan (WS–WBAX) complexes have been evaluated. The results confirmed that WBAX samples with larger Mw and branching degree (HWBAX) significantly impeded gelatinization process of starch by effectively reducing the amount of water available for starch gelatinization. Both molecular characteristics and additive amount of WBAX samples have an effect on the long-term retrogradation behavior of starch. Rheological properties of WS–WBAX mixed gels (the elastic moduli and shear viscosity) increased with the increase in additive amount of non-starch polysaccharide. The scanning electron micrographs (SEM) revealed that the microstructures and the cohesiveness of fresh WS–WBAX mixed gels were mainly affected by the amount of WBAX, but hardly by the molecular characteristics of WBAX. The hardness of mixed gels tended to increase over the 14-day storage [157]. Sugar addition has been reported to increase gelatinization temperature and enthalpy, and affects retrogradation and rheological properties of starch, depending on the types as well as concentrations of both sugar and starch. That was interpreted as producing disorder inside starch granules, thereby increasing the gelatinization temperature of starch [158]. Xylitol was more easily hydrated by water and reduced the available water in the system and acted as anti-plasticizing agent, which caused retardation in gelatinization of wheat starch, leading to higher pasting temperatures. The interaction between xylitol and starch chains stabilized in the amorphous and/or the crystalline of starch granules, which needed greater energy to break during gelatinization [159]. 

## 5. Cellulose-Based Gels

Cellulose is the most known natural polymer originating from varied bioresources and present in large amount which can be separated in bulk quantities from different vegetal sources such as forest trees and cotton-type fibers, lignocellulose waste, including paper waste, and agro-waste (e.g., wheat straw, rice husk, coconut husk, corn cobs, banana peels, sugarcane bagasse, cassava bagasse). The properties featured by cellulose-based materials are dependent on the originating resource, and methodologies applied for processing and alteration. The large potential for designing products from cellulose derived from varied biomass resources suitable for variate domains of application became more and more significant to meet societal needs for advanced and sustainable goods. 

Cellulose, the most versatile polysaccharide synthesized in nature, generates a large range of structural appearances (certain patterns, physical conformations) through self-association or self-ordering processes, and can also undergo the last-mentioned interactions upon moving toward a hydrogel-type structure which opens more ways for exploiting this inexhaustible polysaccharide for obtainment of new materials with different grades of porosity (e.g., aerogels, xerogels) [160,161]. As a non-ionic polysaccharide, cellulose can be also converted into polyelectrolytes by chemical modification (functionalization), the formation of configurable products from cellulose being significantly relevant to the present reality. Processing of cellulose at large industrial scale allows production of a large variety of cellulose-based products such as ethers and esters (known as cellulose derivatives) and precipitated materials (regeneration following chemical processing when fibers, films—nanosheets, food casing, membranes, sponge-like materials can be obtained). 

Cellulose-based gels are three-dimensional hydrophilic polymer networks obtained through various ways including non-covalent bonding (called *physical cross-linking*) and/or covalent bonding (called *chemical cross-linking*) when a variety of monomers or ions is used and infiltrated with solvents (e.g., water, organic solvent/water mixture, ionic liquid). These materials can find sustainable applications related to environmental protection (e.g., water treatment, absorption/adsorption and separation—a good example here is suitability for oil spillage or organic pollutants cleanup) [162,163,164,165,166,167,168,169], thermal protection (may impart insulation properties) [170,171,172], biomedical purposes [173,174,175,176,177,178] (e.g., drug delivery, cell culture, biosensors—may exhibit both self-healing and stimuli-responsive properties), as precursors for making carbon aerogels [179,180] and as carriers of metal nanoparticles and metal oxides [181,182,183]. 

Depending on the solvent medium (as above-mentioned) and employed drying methodology (e.g., solvent evaporation drying method; supercritical drying method using alcohol, acetone, or CO_2_; vacuum freeze-drying method), cellulose-based gels can be:

- *Hydrogels*—which are formed through formation of cross-linked networks using water as the solvent medium. Given the abundance of the functional groups in their unique structure, they present peculiar physical-chemical properties and excellent mechanical properties. Cellulose hydrogels are successfully applied in numerous applicative domains, including wastewater treatment, energy conservation, and restorative therapies [184,185,186,187,188,189,190,191,192] and can be obtained from different agro-waste biomass sources through an effective strategy for cellulose regeneration which involves employment of appropriate chemical pretreatments and bleaching.
- *Aerogels*—which are porous solid materials obtained without use of cross-linking agents in their preparation process which involves three stages: (1) dissolution/dispersion of cellulose or cellulose derivatives, (2) sol-gel process with formation of cellulose-based gel, and (3) drying with preserving a three-dimensional stable porous structure by intra-molecular and inter-molecular physical cross-linking of hydrogen bonds from cellulose structure. Aerogels present an increased permeability, a great specific pore-size, greater density, and proper robustness as mechanical performance making them suitable for a diverse range of applications [193]. Related to the above-mentioned, it is of real significance the relative ease of applying alteration of cellulose structure through chemical processing [194] in order to enhance the durability and peculiarities conferred by the structure of resulted aerogel-type materials (e.g., oxidation using TEMPO).
- *Xerogels*—which are obtained by applying evaporation drying method, which reduces gels’ wetness, presents increased permeability, a great specific pore-size, and a structure similar to aerogels obtained through drying using supercritical solvents [195,196,197,198].

An effectively used way to produce cellulose-based aerogels is to first prepare a cellulose solution with one-component, non-aqueous and non-derivatizing cellulose solvents (substituted pyridinium and imidazolium salts known as ionic liquids ILs). Cellulose and wood can be dissolved in ILs, then resulting solutions (which may still contain non-dissolved cellulose fragments) can form gels by adding a polar anti-solvent (water). Usually, ILs may disrupt the intermolecular hydrogen bonds network from cellulose with maintaining its initial macromolecular structure without degradation. Afterwards, the resulting porous structures can be settled by replacing the liquid with a supercritical solvent. 

Ionic liquids are largely employed as media for the dissolution of polysaccharides oriented toward gel-type materials which are formed through including these green solvents in the polysaccharide network matrix. Ionic liquid-cellulose gel materials (called cellulose ionogels) are a relatively new type of materials that combine cellulose as continuous solid phase component (called matrix) and ionic liquid as dispersed liquid phase [199,200]. Such macromolecular gel materials can find sustainable applications [199] such as catalysis, energy, electronics, medicine, food, cosmetics, and so on, given their versatile potential to be designed through creating new functional features at every component level (cellulose, ionic liquid, and any employed functional additives). Such ionogels can be classified as physical and chemical ionogels, depending on the nature of the interactions (called also involved gelation mechanisms). 

Chemical ionogels are structurally more permanent three-dimensional networks formed by interactions such as covalent bonds (cross-linking). In physical cellulose ionogels, interactions between the cellulose and the ionic liquid are non-covalent (hydrogen bonding, hydrophobic interactions, or van der Waals forces, mainly), all these leading to a self-assembly process with formation of a three-dimensional network. These physical ionogels are easily obtained, the most important part of this process being the dissolution of cellulose in the ionic liquid and the gelation mechanism of the resulting cellulose-ionic liquid solution. Appropriate combination of polysaccharides (as bio-renewable materials) and ionic liquids ILs (as environmentally friendly solvents) may represent an effective, sensitive, green method to prepare novel gel polymer electrolytes (ionogels) applicable for sustainable electrochemical devices. Such an example are the chemically cross-linked chitosan-cellulose ionogels obtained via two-step method in ionic liquid [201] that showed good self-healing ability, stability under testing high temperature values and harsh mechanical conditions, as well as high ionic conductivity, all of these making them suitable for obtainment flexible capacitors. 

Valuable sustainable materials (e.g., composite films—cellulose nano-sheets with good optical properties and gel materials with enhanced biocompatibility and suitability for tissue engineering) can be produced from cellulose solutions by employment treatments such as interchange of dissolution media and regeneration strategies. Cellulose can be precipitated through regeneration from its solution with ionic liquid using water as anti-solvent when a particular range of structure patterns with the same kind of morphology at the microscopic level can result. During these treatments, cellulose substrates undergo some structural changes expressed by crystalline transitions evidenced by X-ray diffraction investigation (cellulose I crystalline allomorph was transformed into cellulose II crystalline allomorph). Generally, the dissolution of cellulose originating from different biomass sources in ionic liquids can be an environmentally friendly alternative to conventional pre-treatment methods. This process can enhance further enzymatic reactions with benefits through upgrade of the release of reducing sugars after hydrolysis as both reaction rate and yield [202]. 

Preparation of polysaccharide ion gels with ionic liquids was comprehensively presented [203,204,205,206,207]. The method involved dissolution of polysaccharides derived from different type substrates (including cellulose) with ionic liquids, mainly 1-butyl-3-methylimidazolium chloride ([BMIM][Cl]). The resulting gels can be well-characterized by using suitable analytical methods and can generate value-added materials by means of exchange with other disperse media and regeneration which can be applied as effective chemical tools for sustainable transformation of cellulose materials. 

**Figure 3:**
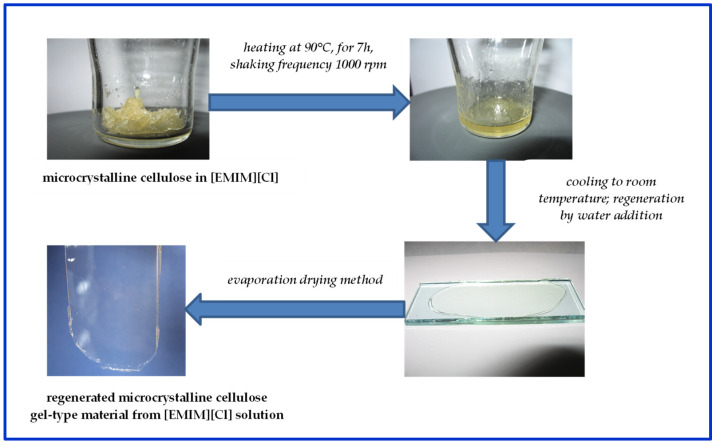
Microcrystalline dissolution in ionic liquid and its regeneration as transparent gel material from solution (re-drawn with permission from [208]).

A previous study [208] investigated dissolution in ionic liquid ([EMIM][Cl]) of different cellulose substrates including microcrystalline cellulose, and celluloses extracted from wood free of extractives—a softwood cellulose (separated from fir tree *Abies alba*) and a hardwood cellulose (separated from beech wood *Fagus sylvatica*)—when gel type materials resulted (as shown in Figure 3 where photos are provided of microcrystalline cellulose sample) as films with transparency by casting the cellulose solutions onto glass plates, followed by their regeneration from solution using water as a coagulant solvent, being safe, environmentally benign, and inexpensive [209].

**Figure 4:**
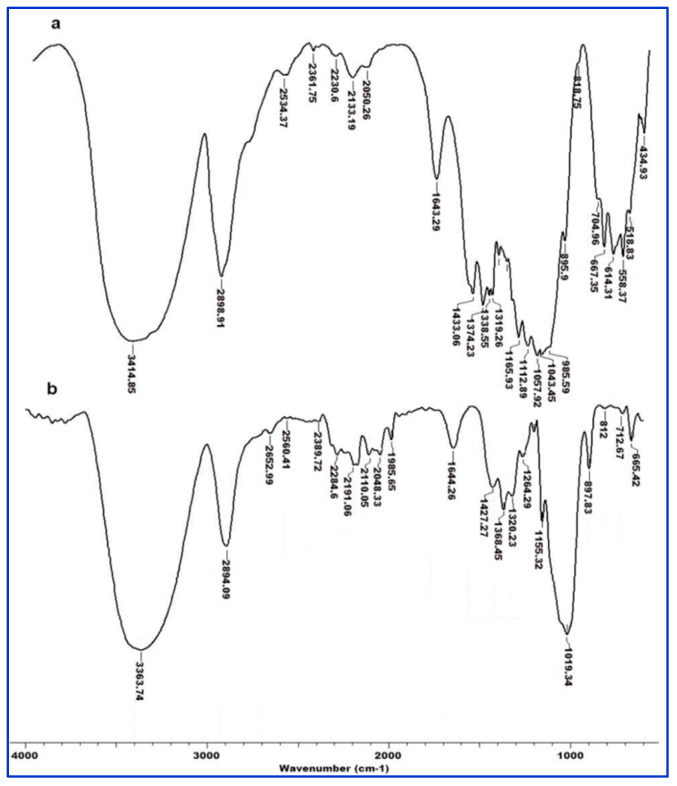
FTIR spectra of microcrystalline cellulose (**a**) and microcrystalline cellulose regenerated from ionic liquid solution (**b**).

**Figure 5:**
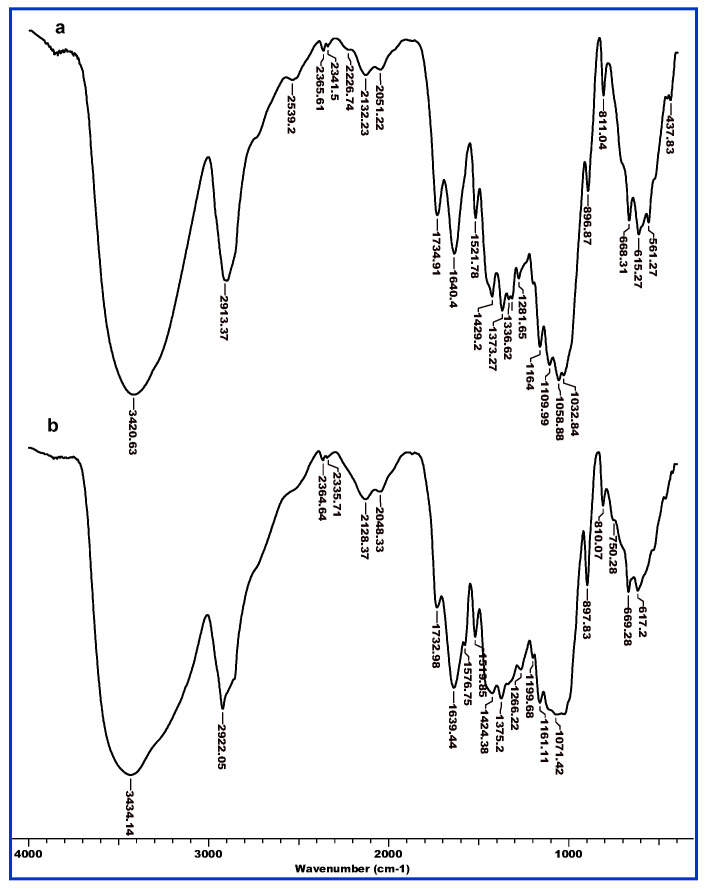
FTIR spectra of wood cellulose from softwood species—fir tree: initial (**a**), and regenerated from ionic liquid solution (**b**).

**Figure 6:**
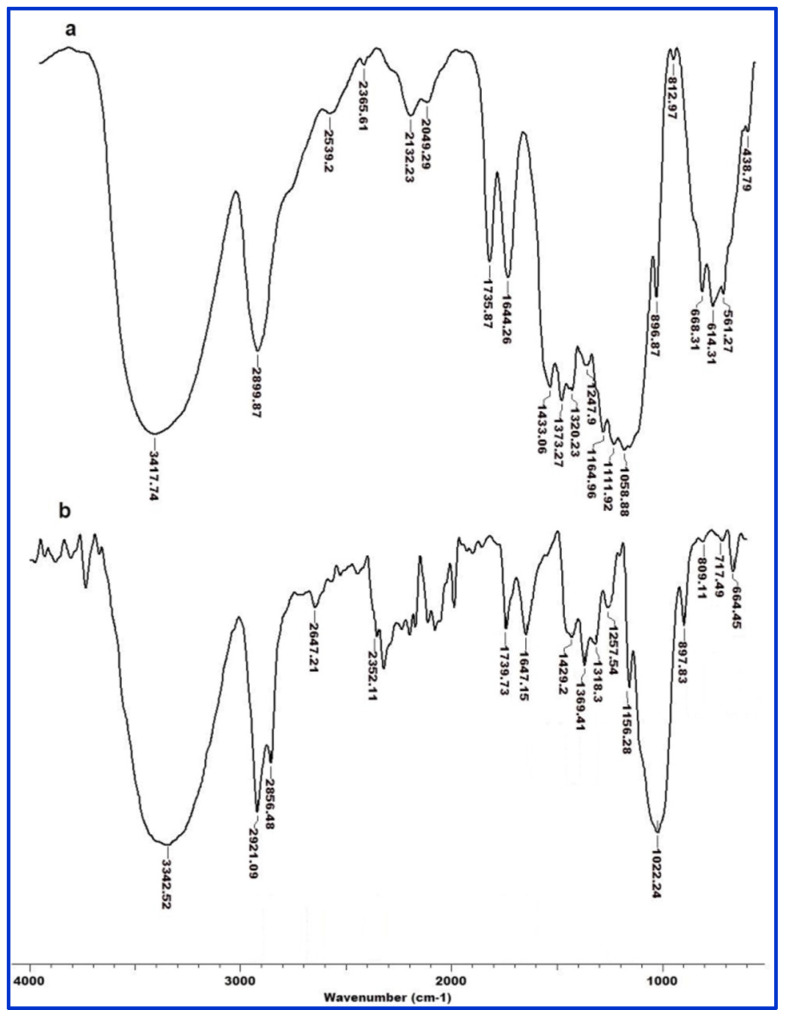
FTIR spectra of wood cellulose from hardwood species—beech tree: initial (**a**), and regenerated from ionic liquid solution (**b**).

Structural investigation of initial cellulose substrates and those regenerated from ionic liquid solution was performed using FTIR spectroscopy (spectra shown in Figure 4, Figure 5 and Figure 6), with evidence of changes in the crystalline part of cellulose. These are expressed by the absorption bands evidenced at 1430 cm^−1^ and 897 cm^−1^, which are highly susceptible in relation to the crystalline part of cellulose structure and employed to calculate the crystallinity index or lateral order index (LOI) as ratio A_1430_/A_897_ [210]. From spectral data, two indexes using ratios of infrared absorption bands can be evaluated: (1) the total crystallinity index (TCI) expressed by A_1372_/A_2900_ ratio [211], and (2) hydrogen-bond intensity (HBI) expressed by A_3308_/A_1330_ ratio [212]. These parameters given in Table 2 are closely related to the crystalline cellulose component in the substrate and the extent degree of regular intermolecular structure.

By adding an anti-solvent (e.g., water) into the cellulose/ionic liquid mixture, a rearrangement of intramolecular hydrogen bonds between the cellulose chains occurred upon destruction of the native cellulose I crystal lattice [213], a fact evidenced by the increased values for HBI in regenerated cellulose substrates. This is also associated with the tendency to stabilize a different conformation of the cellulose macromolecular chains which not only shrink, but also aggregate, precipitate, and regenerate. The TCI values significantly decreased for both cellulose substrates regenerated from ionic liquid solutions (see Table 2). This may be related to the structural transformation suffered by cellulose substrate when some of its crystalline part was transformed into amorphous form. Thus, cellulose dissolution in ionic liquid influenced both the crystalline structural part (well-ordered domains from cellulose structure) and the extent degree of regular intermolecular structure in cellulose substrate. 

## 6. Alginate-Based Gels

### 6.1. General Considerations

Alginates, water-soluble salts of alginic acid, are a group of algal anionic polysaccharides, easily available from renewable resources such as brown seaweed. Most of their applications are in the in medical and bioengineering fields due to their biocompatibility, low toxicity, and rapid gelation in the presence of cations, commonly calcium cations (Ca^2+^) [214], as well as transition divalent cations (Mn^2+^, Co^2+^, Cu^2+^) [215] or various polyvalent ions (Ca^2+^, Zn^2+^, Al^3+^, Mn^2+^, Sr^2+^, Cu^2+^, and Ba^2+^) [216]. 

**Scheme 6:**
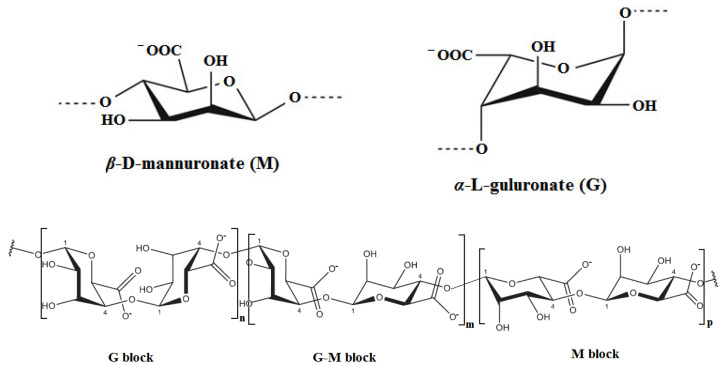
Alginates mer units and polymer structure.

Basically, alginates are linear copolymers consisting of homopolymeric blocks of (1→4)-linked *β*-D-mannuronate (M) and *α*-L-guluronate (G) units (Scheme 6), covalently linked in different sequences. The homopolymeric blocks can be made of consecutive G units (G-blocks), consecutive M units (M-blocks) or alternating M and G units (MG-blocks) [217]. Component G, α-L-guluronate, is the C-5 epimer of M, β-D-mannuronate.

Depending on the source, the content in M and G, and their sequence in the polysaccharides differ [218]. Their functions seem to be distinct, as G sequences in alginate copolymers appeared to be involved in the gel formation of alginates in the presence of Ca^2+^ [219]. Ulterior data indicated that Ca^2+^ alginates have an increased the number of G units which markedly enhanced the binding selectivity, while homopolymeric blocks of (1→4)-linked *β*-D-mannuronate (M) and alternating MG blocks exhibited lower selectivity [220,221] and, therefore, their efficient complexation requires high concentrations of polyvalent ions.

Commercially available alginates have molecular weight in the range 32,000–400,000 g/mol and reach the maximum viscosity at pH = 3 when all carboxylate groups are converted into carboxylic acid groups which participate in an extended network of hydrogen bonds. As far as their cytotoxicity is concerned, highly pure alginates (heavy metal-free) caused no relevant inflammatory response when tested in vivo [222]. On the other hand, it was demonstrated that Ca^2+^ released from alginates gels can produce inflammatory reactions [223], while gels made of alginates in other formulations showed anti-inflammatory effects [224,225]. Nevertheless, alginates are good candidates for tissue engineering [226], drug delivery systems, and cosmetics (as anti-wrinkle agent, moisturizing, and UV-screen) [227].

Given the great number of hydroxylic and carboxylate groups distributed along their macromolecular backbone, alginates can be easily modified by various chemical reactions performed either at the hydroxyl groups (oxidation, reductive amination of oxidized alginate, sulfation, copolymerization, binding β-cyclodextrin) or at the carboxylic ones (esterification, amidation, Ugi four component reaction) [228]. Despite the wide variety of chemical methods of modification, alginate derivatives are not yet commonly used. 

### 6.2. Alginate Gels 

#### 6.2.1. Reversible Alginate Gels

**Scheme 7:**
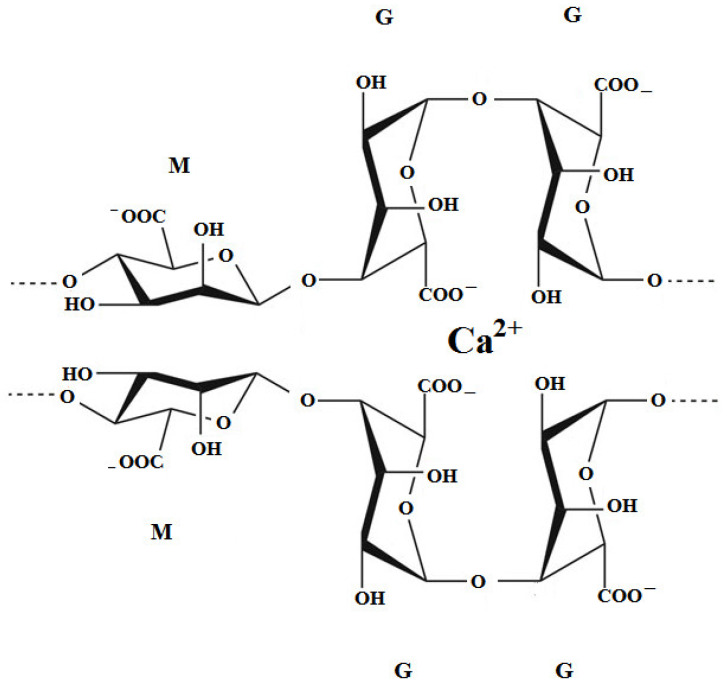
Structure of reversible Ca-alginate gels (ionic interactions).

Sodium alginate solutions can readily form reversible gels in the presence of calcium chloride. The physical hydrogel occurs by the ionic interactions between Ca^2+^ and carboxylate groups originating mainly from vicinal G units (Scheme 7), where the maximal coordination was achieved. They are reversible gels due to their short-term stability; when calcium cations start to migrate from the gel structure, the ionic interactions become weaker and weaker, until the entire supramolecular assembly disintegrates. 

Other coordination reagents have been studied, namely sodium hexametaphosphate, calcium sulfate, and calcium carbonate [219] and it was concluded that gelation occurred under the most favorable conditions (control of gel properties) only in the presence of the reagent that allowed the lowest gelation rate. This condition is fulfilled by calcium carbonate which has a very low solubility in water, at pH = 7. When the pH decreased, calcium cations were gradually released in the alginate solution and initiated the controlled gel formation.

The presence of polyols had the same effect: they reduced the alginate gelation rate by hindering the ionic complexation of carboxylate groups with calcium cations [229].

Some rheology studies on the non-linear viscoelastic properties of alginate gels indicated a strain-hardening behavior during large deformation, similar to many other biopolymers, but the degree of strain-hardening depended on gel composition [230]. This phenomenon was attributed to the deformation of rod-like segments where physical crosslinked bridges have been formed in the gel structure. The proposed model has been validated by experimental results from torsion and compression, and included the stretching of Gaussian network chains additionally to the stress caused by the deformation of rigid cross-linking bridges.

#### 6.2.2. Stable Alginate Gels

Irreversible alginate gels can be obtained by chemical or photochemical cross-linking when permanent covalent bonds are formed between alginates and the cross-linking reagents.

##### Ionotropic Alginate Gels

Gels made of alginate having various amounts of guluronic fraction (high or low G content, HG and LG, respectively) and transition divalent cations (Mn^2+^, Co^2+^, Cu^2+^) have been prepared and characterized by the means of rheology and spectral-mechanical analysis (recording the complex shear modulus G*= G′+ j·G′′ as a function of the shearing angular frequency ω (10^−3^–100 rad/s) in decreasing mode and controlling the normal force close to zero) [215]. Experimental data indicated that gels made of Cu-HG, Cu-LG, and Co-HG displayed a viscoelastic behavior, as the elastic contribution was higher than the dissipative one, while gels Mn-HG, Mn-LG, and Co-LG behaved like common entangled polymers. Cu-alginates having a fibrillar structure were found to possess high rubbery rigidity and an apparent infinite relaxation time. Mn-alginate gels structure proved to be more complex regardless the G:M ratio. Co cations produced gels of both types depending on the G:M ratio; Co-HG had a simple fibrillar structure, but Co-LG showed a more complex structure.

When employed in very low concentrations, Mn^2+^, Co^2+^, and Cu^2+^ initiated the alginate gelation at higher rates than Ca^2+^ and the rate depended on the temperature, which is directly related to the gelation mechanism; at low temperatures (5 °C), the intra-molecular cross-linking was prevalent, and when temperature was increased up to 50 °C, the structure changed as the interactions turned into irreversible inter-molecular cross-linking. Upon higher concentration of divalent cations, it was possible to engage a higher number of G and M units, which allowed a more complex evaluation of the viscoelastic behavior of gels, and the secondary structures depended not only on the nature of the cation, but on the G:M ratio as well. For example, Cu-alginate gels with high content in M or G showed a monodisperse rod-like secondary structure, similar to Ca-alginates [231], Mn^2+^ cations lead to gels structure with various junction zone multiplicities [232], while Co- and Zn-alginate gels formed structures depending on the M:G ratio as follows: M-rich alginates showed a complex secondary structure, but gels with high amounts of G displayed a simple rod-like structure [233]. 

**Figure 7:**
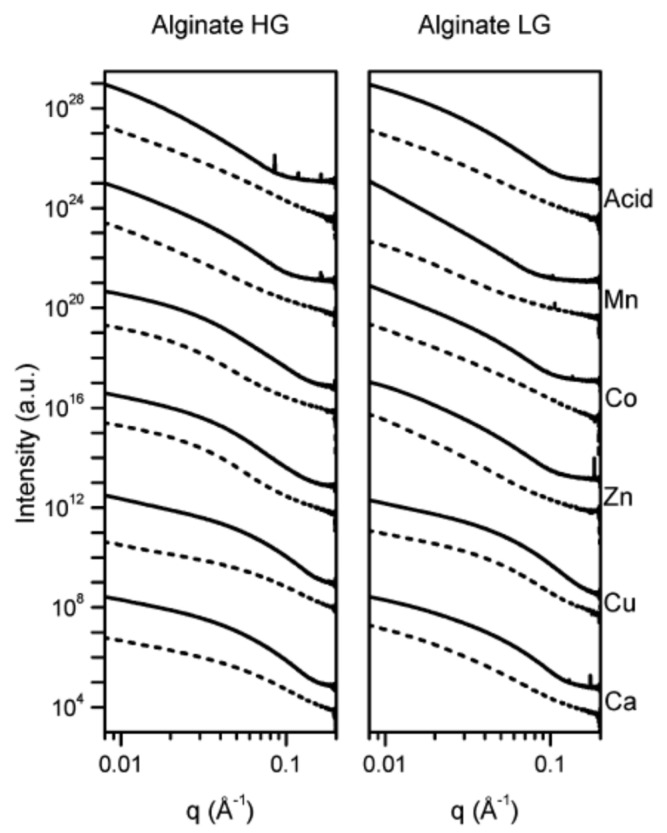
SAX profiles for aerogels (solid lines) and hydrogels (dashed lines) obtained from alginates with high G (HG) or high M/low G (LG) units’ content. Reprinted with permission from [233] (Copyright 2012, American Chemical Society).

The supramolecular assembly of macromolecular chains of alginates is based on the cooperative associations of vicinal chains and depends on both G/M ratio and the nature of the cation. SAX spectrometry data recorded for aerogel and hydrogels prepared from alginates with high content in G units (HG) or in M units (LG) (Figure 7) allowed a further discrimination of structures at the nanometric scale. Thus, based on the value of the scattering exponent (*α*), it was demonstrated that hydrogels having *α* ≈ 1 and containing Ca, Cu, Zn and Co (for Ca-LG, Ca-HG, Cu-LG, Cu-HG, Zn-HG, and Co-HG) displayed a fine fibrillar morphology, while hydrogels for which *α* > 2, namely H-HG, H-LG, Mn-LG, Mn-HG, Zn-LG, and Co-LG, showed a more complex morphology.

**Figure 8:**
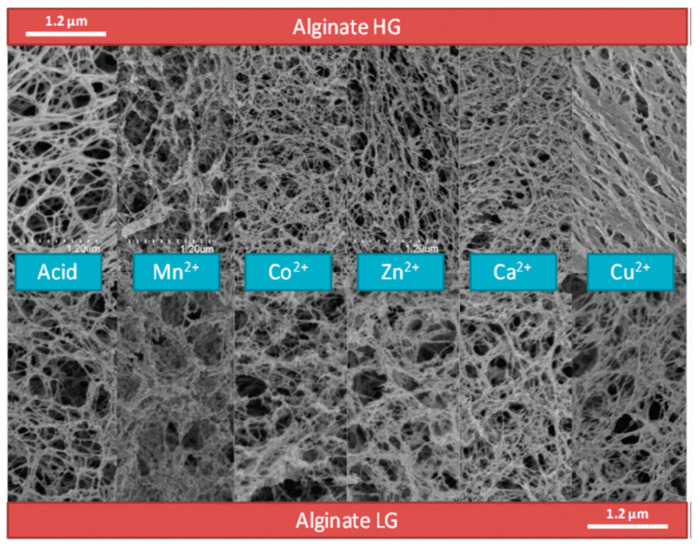
SEM micrographs of HG and LG metal-alginates aerogels. Reprinted with permission from [233] (Copyright 2012, American Chemical Society).

Furthermore, Zn- and Co-HG exhibited fibrillar structures, whereas the corresponding LG hydrogels showed junctions with multiple morphologies, as result of the amount of G/M units in the structure of the initial alginates. At the same time, Cu^2+^ interacts strongly with both M and G units, yielding fibrillar morphology regardless of the G/M ratio, whereas Ca cations displayed an affinity for G units to form fibrillar structures. These conclusions were supported by data collected from SEM micrographs (Figure 8) where the morphology of metal-alginates aerogels has been illustrated.

All samples displayed porous tridimensional structures with interconnected pores, where slight differences in network density and fiber roughness were noticed. 

In conclusion, the macroscopic properties of transition metal cations-alginate gels are the result of structural peculiarities as the fibrillar structures entailed a rubbery behavior, while the complex structure, specific to entangled polymer chains, caused flowing behavior. 

Polyvalent ions (Ca^2+^, Zn^2+^, Al^3+^, Mn^2+^, Sr^2+^, Cu^2+^, and Ba^2+^) cross-linked with alginate gels have been structurally investigated by the means of solid-state NMR spectroscopy, namely ^1^H- and ^13^C-NMR, ^13^C CP/MAS NMR, ^23^Na 3Q/MAS NMR, 2D ^27^Al TQ/MAS NMR, and 2D ^27^Al 3Q/MAS NMR, and density functional theory (DFT) calculus [216]. Experimental data confirmed the homogeneous distribution of cross-linking cations in the alginate gel microbeads and the high degree of ion exchange during cross-linking. A certain phase separation has been revealed in alginate gels, caused by the preferential localization of residual water molecules (5–10% wt) in the region of M units and carbonyl moieties, both phases coexisting and differing in their segmental motion. The mobile segments of alginate gels in hydrated domains are scarce, but they have high-amplitude motions (average fluctuation angle up to 30°). 

**Figure 9:**
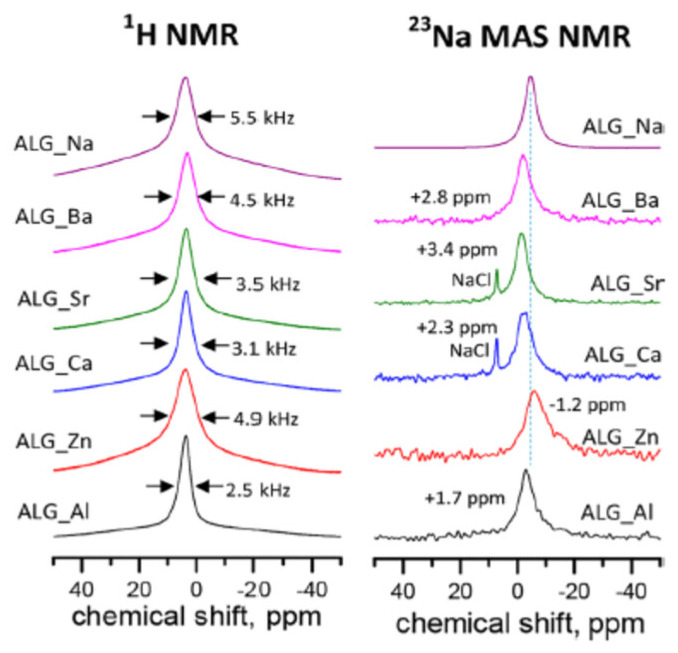
^1^H NMR and ^23^Na MAS NMR spectra of mono- (Na^+^) and multivalent ions alginate gels. Reprinted with permission from [216] (Available online: ttps://pubs.acs.org/doi/full/10.1021/acs.biomac.7b00627. Accessed on 14 November 2022. Further permission related to the material excerpted should be directed to the ACS).

It was possible to evidence the degree of ion exchange (Na versus polyvalent cations) by the means of ^23^Na MAS NMR spectroscopy and by comparing the intensity of signals attributed to the corresponding ions (Figure 9). The ion exchange was almost complete (residual Na^+^ 3–7%), but uronic units showed higher affinity for divalent cations than for the trivalent ones. The associated ^1^H NMR spectra indicated that alginate gel flexible segments contain water molecules entrapped in the tridimensional structures, while the rigid segments do not, and therefore limit their further diffusion. 

**Figure 10:**
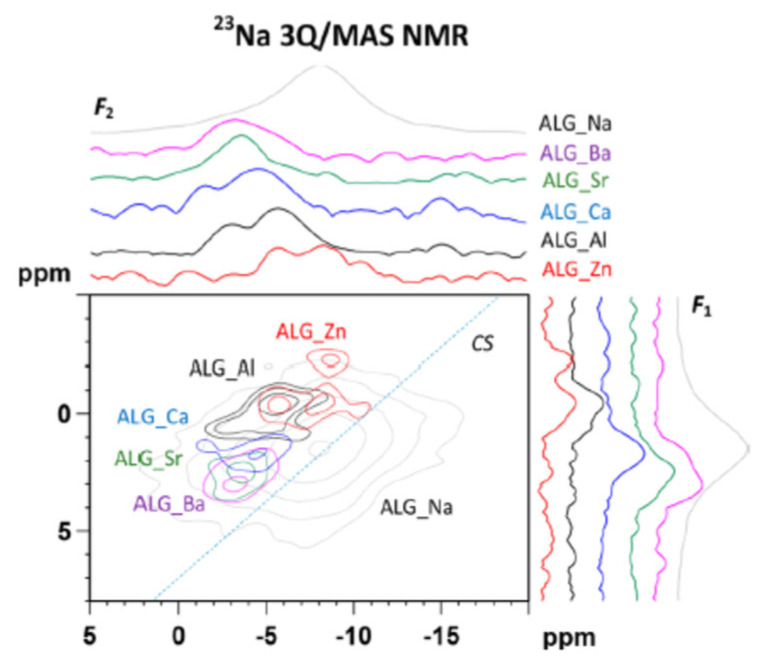
2D ^23^Na 3Q/MAS NMR spectra of mono- (Na^+^) and multivalent ions alginate gels. Reprinted with permission from [216] (Available online: https://pubs.acs.org/doi/full/10.1021/acs.biomac.7b00627. Accessed on 14 November 2022. Further permission related to the material excerpted should be directed to the ACS).

Employing ^23^Na 3Q/MAS NMR it was possible to obtain spectra with enhanced resolution that indicated significant changes occurred in the alginate supramolecular structures during crosslinking in the presence of multivalent cations (Figure 10). Thus, it was suggested that areas with distinct local geometry may coexist in the vicinity of the residual Na cations that are rather homogeneously dispersed in the new alginates.

It was also demonstrated that the amount of M units in alginates and the nature of cation strongly influenced the microstructure of gels. Thus, the regularity of polymer segments decreased with the decreasing radius of cross-linking cations: Ba-, Sr- or Ca-alginate gels had a highly ordered structure, while Zn- and Al-alginates showed significantly low-ordered structures, mainly in M units, as G units remained in well-ordered structures. Entangled macromolecular chains in M segments favored the enhanced hydrogen bonding, suggesting that M blocks and M-rich copolymer blocks promoted the self-assembly of alginate gels.

**Figure 11:**
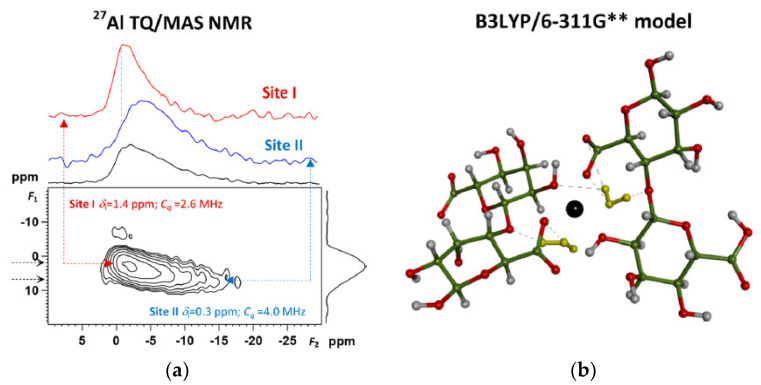
(**a**) 2D ^27^Al TQ/MAS NMR spectrum of Al-alginate; (**b**) computational model of Al-alginate—the octahedral arrangement (yellow lines) of the ligands surrounding Al^3+^ (the black circle). Reprinted with permission from [216]. (Available online at: https://pubs.acs.org/doi/full/10.1021/acs.biomac.7b00627. Accessed on 14 November 2022. Further permission related to the material excerpted should be directed to the ACS).

In the case of Al-alginates, two distinct six-coordinated Al^3+^ cross-linking centers with octahedral geometry and different local dynamics have been evidenced. Thus, using 2D ^27^Al TQ/MAS NMR spectrometry it was possible to evidence the presence of two Al^3+^ centers (two isotropic positions) and represent the projections extracted for the two resolved sites (Figure 11a). Associated data on the local motion of ligands and preferential localization of residual water molecules (5%) suggest the existence of two types of cross-linking centers with comparable spheres of ligands, but significantly different local dynamics.

Based on DFT calculations, the computational model of Al-alginate was obtained (Figure 11b) and it illustrated the octahedral conformation of the ligands coordinated by an Al^3+^ central cation, showing that the ligand field around the crosslinking site is asymmetric.

SEM images confirmed that ion-alginate gel beads differ in morphology (particle size, shape, and surface). Alginates cross-linked by divalent cations formed spherical particles, with smooth and homogeneous surface when large cations like Ba^2+^ and Sr^2+^ were employed; Al-alginate gel particles were significantly flatty and with porous surface morphology.

An interesting study focused on the effect of the solvent, and mixtures of water and alcohols, on the solubility and properties of Ca-alginate gels [234]. It was concluded that the addition of low to moderate amounts of ethanol (no more than 15%) increased the intrinsic viscosity of gels. The performance of the solvent is lower at higher ethanol content (20%) when the viscosity decreased. The same tendency was observed for the moduli and the stress at break. SAXS and TEM data indicated gels acquired an increasingly rough surface and a heterogeneous network (regions of precipitated polymer) when moderate-to-high amounts of ethanol (15–24%) have been employed. The weakness of the Ca-alginate gel was attributed to the poorly crosslinked macromolecular chains.

Other characterization methods, such as isothermal thermogravimetry in a kinetic mode associated with the time-resolved infrared spectroscopy, were used to confirm the role of solvent, especially when the solvent was water, in the formation and properties of polysaccharide gels [194].

##### Gelation by the Reaction with Bi- and Multi-Functional Cross-Linkers

Adipic dihydrazide is the reagent used for the gelation of chemically modified alginate, namely oxidized alginate [222]. Alginate was selectively oxidized in the presence of sodium periodate, under mild conditions, when vicinal (C2-C3) carbonyl groups were formed, according to the mechanism reported in the literature [235]. The gelation mechanism occurred through reductive amination that was initiated by the presence of the dihydrazide, when the aminic groups reacted with the carbonyl ones in the presence of poly(ethylene glycol).

Gelation of alginates can be performed in the presence of glutaraldehyde when an acetal bridge is formed with the hydroxyl groups in alginate [236]. 

Glucono-δ-lactone (GDL) is another cross-linker employed in the gelation of alginates, mainly when alginates are in different combinations with other biopolymers, such as proteins, or even other polysaccharides. For example, sodium casein-alginate composite gels have been prepared in the presence of GDL with various amounts of alginate (0.2, 0.4, and 0.75%, respectively) [237]. When alginate was combined with another polysaccharide, namely partially deacetylated gellan, the complex structures obtained by gelation in the presence of GDL yielded interpenetrating network gels [238]. Their properties can be tuned by controlling the alginate/gellan ratio.

In a combined approach, GDL has been used along with Ca carbonate to obtain highly ordered, highly porous (interconnected pores), biodegradable, hemo- and cytocompatible alginate hydrogels loaded with vitamin D3 [239].

Alginate irreversible gels with improved stability and mechanical properties have been prepared by employing multi-functional cross-linkers, such as poly(acrylamide-co-hydrazide) [240].

Photochemically cross-linked alginate gels are irreversible gels obtained using a previously chemically modified alginate bearing photochemical reactive groups, namely methyl acrylate moieties [241,242,243]. The methacrylated alginate macromers and the corresponding photochemically cross-linked hydrogels showed reduced cytotoxicity towards primary bovine chondrocytes or osteoblasts, which recommends these materials for medical and tissue engineering applications.

##### Alginate Gels by Non-Conventional Methods

Some non-conventional methods of alginates gelation have been reported as well, but they were applied to native alginates [244].

Cryotopic gelation (cryogelation) is a three-step procedure (non-deep freezing of the solution of monomers or precursors, storage in frozen state, and thawing) that allows the manufacture of cryogels with a large fraction of interconnected pores of micrometric size (10–100 μm) [245]. It can be performed in different procedures: cation-free (applied for native alginates or alginates in various formulations with PVA, gelatin, hyaluronic acid, etc.) or ionotropic cryogelation (in the presence of calcium salts that showed increased solubility in water along with the decreasing temperature, such as calcium butyrate, pentanoate, succinate, or glycerophosphate) [246].

Non-solvent induced phase separation (NIPS) is also known as immersion precipitation and was employed to obtain lyogels which can subsequently harden by the advanced removal of the solvent [247]. The gelation mechanism of NIPS may be attributed to the contraction of hydrophilic alginate macromolecular chains upon the addition of a non-solvent. At the same time, lateral associations of alginate macromolecules occurred, and they are expected to contribute to reducing the number of hydrophilic groups exposed to the non-solvent [248].

Carbon-dioxide-induced gelation takes place when a suspension of metal carbonate or hydroxycarbonate (Ca, Sr, Ba, Zn, Cu, Co, Ni) in a sodium alginate aqueous solution is submitted to pressurized CO_2_ (30–50 bar) at room temperature. This protocol can be used for a wide range of alginate blends with other biopolymers or water-soluble synthetic polymers when alginates are considered in the polymer matrix for the incorporation of insoluble microparticles [249,250]. At the end of the process, significant amounts of CO_2_ remained dissolved in the aqueous phase of the gel. 

Other non-conventional gelation methods for alginates have been investigated: gelation in the presence of carboxylic acids (oxalic, maleic, tartaric, glutaric, and citric acid); water electrolysis in the aqueous solution of sodium alginate [251]; photochemical reduction of Fe^3+^ to Fe^2+^ from Fe-alginate gels in the presence of 2-hydroxybutyric acid and CaCl_2_, when an ion exchange occurred (Fe^3+^ has a higher affinity toward alginate than Fe^2+^, so it is substituted by Ca^2+^ upon reduction) [252]; crosslinking the solid sodium alginate with 1,6-hexamethylene diisocyanate (HDI) in dry benzene as inert solvent ratio [253]. 

In conclusion, alginate gels are highly versatile materials despite their rather simple structure. The presence of the two distinct monomer units allows controlled morphology and tunable properties. Depending on the gelation method and mechanism, specific supramolecular structures can be evidenced and studied by a wide variety of characterization methods. Thus, it was possible to reveal the particular structure–properties relationship. The main structural peculiarities are the result of the alginates’ secondary and tertiary structures, their interactions with the solvent and/or non-solvent molecules and with other components, and processing parameters (temperature, pH, pKa). Either alone or in complex formulations along with other natural or synthetic polymers, alginates allow gels with characteristics that recommend them for various applications in medicine, healthcare, biomedical engineering, industrial water purification, etc. 

## 7. Conclusions

Polysaccharide gels proved to be of high interest from both theoretic and practical point of view due to their availability and abundance from renewable resources, structural and functional properties, and wide range of applications. The origin, processing, and further modification strategies influence their level of performance, and thorough investigations confirmed the possibility of tailoring the properties of these versatile materials according to the requirements of intended applications.

The present survey offers insights into the structural peculiarities of a few polysaccharide gels, namely chitosan, dextran, cellulose, starch, and alginates, in correlation with their state (native or modified), formulation, and processing parameters, the structure-properties relationship, and using modern methods or combined approaches of characterization. These polysaccharides have been selected on the base of their theoretic relevance, applicative potential, and volume of publications reported in the last decade, criteria that indicate them as highly engaging for the R&D scientists.

This study confirmed that the structural characteristics of each group of gels are strongly related to their chemical composition, as functional groups—inserted into or pendant to the macromolecular chains—determine the secondary and tertiary structures, in close correlation with the vicinal macromolecules, surrounding medium (mostly water molecules, free or entrapped in different polyhedral conformations), or partner molecules/ions (aminoacids, mono- or multivalent cations) able to coordinate supramolecular assemblies. 

Considering the structure–properties relationship as defining the level of performance and range of applications, many researchers have focused in recent years on improving the characteristics of these gels, or even on manufacturing materials with novel properties. Designing and testing new formulations opened perspectives for high-tech applications (sensing, energy storage, environmental applications). Thus, a wide variety of formulations emerged (blends, micro-/nanocomposites) and were conditioned under various forms (films, nanoparticles, beads, etc.), where polysaccharides were combined with each other or with other natural polymers (e.g., animal proteins such as gelatin and silk fibroin; vitamins; vegetal oils, etc.), synthetic polymers, micro-/nanoparticles (graphene and graphene oxide) and/or fibers (bacterial cellulose nanofibrils), metal cations and metal oxides. Associated with advanced and complex means of characterization, this new trend may offer solutions to some problems that have been raised in practice (processability, chemical and physical stability, affinity toward specific media, biodegradation).

This domain is very active and progressing at a high rate, which is promising for new materials and new horizons.

## Figures and Tables

**Table 1:** Some novel formulations of polysaccharides and other components.

Formulation	Application/ Main Property	Observations	Ref.
Alginate and gelatin	Injectable self-crosslinking hydrogels	Previously, alginate was selectively oxidized in the presence of sodium periodate to yield dialdehyde alginate	[38]
Xanthan, guar and *Mesona chinensis* Benth gums, and pea starch	Improved viscoelasticity, hardness, chewiness, and thermal stability	XG, GG, and MCG enhanced pasting stability and gel properties. Compact structure in PS-hydrocolloid was due to the hydrogen bonds. PS-MCG gels showed enhanced deformability, hardness, and thermal stability.	[39]
Gum arabic (GAR), octenyl succinic anhydride modified starch (OSA), water soluble soy polysaccharides (WSSP), and gelatin (GEL)	Polymer matrix for the antioxidant ascorbyl palmitate (AP) able to provide fast release under controlled conditions and prolonged antioxidant activity	GAR enabled the controlled release of AP, and the prolonged antioxidant activity. OSA enabled the fast release of AP.	[40]
Chitosan and gelatin	Thermosensitive chitosan-gelatin hydrogels intended for biomedical applications were obtained by double crosslinking	The non-toxic hydrogels provided increased resistance time and improved efficacy for the selected drug. The drug release was controlled and the gels protected the drug from clearance along with tears. The in situ fast gel formation was observed at the instillation site.	[41]
Gum arabic/gelatin and water-soluble soy polysaccharides/gelatin	Carriers for water-soluble antioxidant AstaSana astaxanthin (AST) able to provide controlled release and preserve the antioxidant activity	Release of AST from the WSSP-based carrier was slower than from the GAR-based film. Native starch behaved as a binder and can be used for retarded release.	[42]
κ-Carrageenan and a low-transition temperature mixture made of fructose, glycerol, and water	Thermosensitive wholly bio-based physical gels	The thermosensitive gelation was attributed to the formation of aggregated H-bonded helices composed of κ-carrageenan chains. SAXS data suggested a more extended conformation leading to an extended physical cross-linking, yielding improved thermal and mechanical stability.	[43]
Polysaccharides and proteins	Functional hydrogels as delivery systems for bioactive ingredients used in food industry	Polysaccharide/protein hydrogels can control the release rate of bioactive ingredients and enhance their bioavailability.	[44]
Proteins and polysaccharides	Edible oleogels fabricated from proteins and polysaccharides as polymer gelators, to be used in food industry as functional healthy solid fat replacers		[45]
Various polysaccharides and synthetic/natural polymers	Drug delivery systems	Enhanced targeting and controlled delivery, low immunogenicity, excellent biocompatibility, readily biodegradable.	[46]
Polysaccharide hydrogels and oleogels made of vegetal oils	Bigels for biomedical applications	Transdermal drug delivery was possible due to their particular properties: mucoadhesion, the ability to control the drug release, the possibility to simultaneously include hydrophilic and lipophilic drugs.	[47]
Polysaccharides	Gels as ingredients and edible, biodegradable, and safe coating materials for various biomedical applications.	They can modulate the intestinal microbial population (limitation of pathogens proliferation; controlling commensal bacteria and probiotics; enhanced host-microorganisms interactions) with beneficial effects.	[48]
Polysaccharides ionogels and various other co-components, natural and/or synthetic	Divalent metal cations based hydrogels for energy storage, sensing, biomedical and environmental applications.	Polymer blends and composites based on polysaccharides and metal and metal oxides nanoparticles, vitamins, bacterial cellulose fibers, β-cyclodextrin, gelatin, graphene oxide, grafted hyaluronic acid, aminoacids, protein nanofibrils, quaternary uranyl salts—gels with new properties for technical applications.	[49]

**Table 2:** Some indexes calculated from spectral data for cellulose substrates, initial and after regeneration from ionic liquid solution (re-drawn with permission from [208]).

Cellulose Substrate	A_1430_/A_897_ (LOI)	A_1372_/A_2900_ (TCI)	A_3308_/A_1330_ (HBI)
microcrystalline cellulose	1.514	1.269	1.502
microcrystalline cellulose after regeneration from solution with ionic liquid	1.332	0.662	5.555
hardwood cellulose	1.738	1.369	0.676
hardwood cellulose after regeneration from solution with ionic liquid	1.106	0.297	5.750

## Data Availability

Not applicable.

## References

[1] Gul K., Gan R.Y., Sun C.X., Jiao G., Wu D.T., Li H.B., Kenaan A., Corke H., Fang Y.P. (2022). Recent advances in the structure, synthesis, and applications of natural polymeric hydrogels. Crit. Rev. Food Sci. Nutr..

[2] Yang X., Li A., Li X., Sun L., Guo Y. (2020). An overview of classifications, properties of food polysaccharides and their links to applications in improving food textures. Trends Food Sci. Technol..

[3] Yang X., Li A., Li D., Guo Y., Sun L. (2021). Applications of mixed polysaccharide-protein systems in fabricating multi-structures of binary food gels—A review. Trends Food Sci. Technol..

[4] Nešić A., Cabrera-Barjas G., Dimitrijević-Branković S., Davidović S., Radovanović N., Delattre C. (2019). Prospect of polysaccharide-based materials as advanced food packaging. Molecules.

[5] Joseph J., Kanchalochana S.N., Rajalakshmi G., Hari V., Durai R.D. (2012). Tamarind seed polysaccharide: A promising natural excipient for pharmaceuticals. Int. J. Green Pharm..

[6] Srivastava P., Malviya R. (2011). Sources of pectin, extraction and its applications in pharmaceutical industry−An overview. Indian J. Nat. Prod. Resour..

[7] Bahú J.O., de Andrade L.R.M., de Melo Barbosa R., Crivellin S., da Silva A.P., Souza S.D.A., Cárdenas Concha V.O., Severino P., Souto E.B. (2022). Plant polysaccharides in engineered pharmaceutical gels. Bioengineering.

[8] Priyan Shanura F.I., Kim K.N., Kim D., Jeon Y.J. (2019). Algal polysaccharides: Potential bioactive substances for cosmeceutical applications. Crit. Rev. Biotechnol..

[9] Xiong Y.H., Zhang L., Xiu Z., Yu B., Duan S., Xu F.J. (2022). Derma-like antibacterial polysaccharide gel dressings for wound care. Acta Biomater..

[10] Shen S., Chen X., Shen Z., Chen H. (2021). Marine polysaccharides for wound dressing application: An overview. Pharmaceutics.

[11] Duceac I.A., Verestiuc L., Dimitriu C.D., Maier V., Coseri S. (2020). Design and preparation of new multifunctional hydrogels based on chitosan/acrylic polymers for drug delivery and wound dressing applications. Polymers.

[12] Zamboulis A., Michailidou G., Koumentakou I., Bikiaris D.N. (2022). Polysaccharide 3D printing for drug delivery applications. Pharmaceutics.

[13] Salave S., Rana D., Sharma A., Bharathi K., Gupta R., Khode S., Benival D., Kommineni N. (2022). Polysaccharide based implantable drug delivery: Development strategies, regulatory requirements, and future perspectives. Polysaccharides.

[14] Duceac I.A., Vereștiuc L., Coroaba A., Arotăriței D., Coseri S. (2021). All-polysaccharide hydrogels for drug delivery applications: Tunable chitosan beads surfaces via physical or chemical interactions, using oxidized pullulan. Int. J. Biol. Macromol..

[15] Chandra N.S., Gorantla S., Priya S., Singhvi G. (2022). Insight on updates in polysaccharides for ocular drug delivery. Carbohydr. Polym..

[16] Yang Q., Peng J., Xiao H., Xu X., Qian Z. (2022). Polysaccharide hydrogels: Functionalization, construction and served as scaffold for tissue engineering. Carbohydr. Polym..

[17] Duceac I.A., Lobiuc A., Coseri S., Verestiuc L. Tunable Hydrogels Based on Chitosan, Collagen and Poly (Acrylic Acid) for Regenerative Medicine. Proceedings of the 2019 E-Health and Bioengineering Conference (EHB).

[18] Malliappan S.P., Yetisgin A.A., Sahin S.B., Demir E., Cetinel S. (2022). Bone tissue engineering: Anionic polysaccharides as promising scaffolds. Carbohydr. Polym..

[19] Duceac I.A., Tanasa F. (2020). Novel chitosan-hydroxyapatite macroporous composites for biomedical applications. Rev. Roum. Chim..

[20] Neto J.R., Copes F., Chevallier P., Vieira R.S., da Silva J.V.L., Mantovani D., Beppu M.M. (2022). Polysaccharide-based layer-by-layer nanoarchitectonics with sulfated chitosan for tuning anti-thrombogenic properties. Colloids Surf. B Biointerfaces.

[21] Schiavi A., Cuccaro R., Troia A. (2022). Functional mechanical attributes of natural and synthetic gel-based scaffolds in tissue engineering: Strain-stiffening effects on apparent elastic modulus and compressive toughness. J. Mech. Behav. Biomed. Mater..

[22] Isobe N., Tsudome M., Kusumi R., Wada M., Uematsu K., Okada S., Deguchi S. (2020). Moldable crystalline α-chitin hydrogel with toughness and transparency toward ocular applications. ACS Appl. Polym. Mater..

[23] Rees D.A., Welsh E.J. (1977). Secondary and tertiary structure of polysaccharides in solutions and gels. Angew. Chem. Int. Ed..

[24] Tako M. (2000). Structural principles of polysaccharide gels. J. Appl. Glycosci..

[25] Tako M. (2015). The principle of polysaccharide gels. Adv. Biosci. Biotechnol..

[26] Tako M., Tamaki Y., Teruya T., Takeda Y. (2014). The principles of starch gelatinization and retrogradation. Food Nutr. Sci..

[27] Cao Y., Mezzenga R. (2020). Design principles of food gels. Nat. Food.

[28] Tako M., Hanashiro I. (1997). Evidence for a conformational transition in curdlan. Polym. Gels Netw..

[29] Tako M., Tamaki Y., Konishi T., Shibanuma K., Hanashiro I., Takeda Y. (2008). Gelatinization and retrogradation characteristics of wheat (Rosella) starch. Food Res. Int..

[30] Gamini A., Toffanin R., Murano E., Rizzo R. (1997). Hydrogen bonding and conformation of agarose in methyl sulfoxide and aqueous solutions investigated by ^l^H and ^13^C NMR. Carbohydr. Res..

[31] Ganesan K., Budtova T., Ratke L., Gurikov P., Baudron V., Preibisch I., Niemeyer P., Smirnova I., Milow B. (2018). Review on the production of polysaccharide aerogel particles. Materials.

[32] Qi X., Tong X., Pan W., Zeng Q., You S., Shen J. (2021). Recent advances in polysaccharide-based adsorbents for wastewater treatment. J. Clean. Prod..

[33] Jin W., Xiang L., Peng D., Liu G., He J., Cheng S., Li B., Huang Q. (2020). Study on the coupling progress of thermo-induced anthocyanins degradation and polysaccharides gelation. Food Hydrocoll..

[34] Usmiati S., Richana N., Mangunwidjaja D., Noor E., Prangdimurti E. (2014). The using of ionic gelation method based on polysaccharides for encapsulating the macromolecules–a review. Encapsul. Prot. Bioact. Compd..

[35] Rinaudo M. (2008). Main properties and current applications of some polysaccharides as biomaterials. Polym. Int..

[36] Tamaki Y., Konishi T., Tako M. (2011). Gelation and retrogradation Mechanism of wheat amylose. Materials.

[37] Tako M., Teruya T., Tamaki Y., Uechi K., Konishi T. (2021). Molecular origin for strong agarose gels: Multi-stranded hydrogen bonding. J. Polym. Biopolym. Phys. Chem..

[38] Resmi R., Parvathy J., John A., Joseph R. (2020). Injectable self-crosslinking hydrogels for meniscal repair: A study with oxidized alginate and gelatin. Carbohydr. Polym..

[39] Rong L., Shen M., Wen H., Xiao W., Li J., Xie J. (2022). Effects of xanthan, guar and Mesona chinensis Benth gums on the pasting, rheological, texture properties and microstructure of pea starch gels. Food Hydrocoll..

[40] Łupina K., Kowalczyk D., Drozłowska E. (2020). Polysaccharide/gelatin blend films as carriers of ascorbyl palmitate–A comparative study. Food Chem..

[41] Song Y., Nagai N., Saijo S., Kaji H., Nishizawa M., Abe T. (2018). In situ formation of injectable chitosan-gelatin hydrogels through double crosslinking for sustained intraocular drug delivery. Mater. Sci. Eng. C.

[42] Łupina K., Kowalczyk D., Kazimierczak W. (2021). Gum arabic/gelatin and water-soluble soy polysaccharides/gelatin blend films as carriers of astaxanthin—A comparative study of the kinetics of release and antioxidant properties. Polymers.

[43] Caprin B., Viñado-Buil G., Sudre G., Morelle X.P., Da Cruz-Boisson F., Charlot A., Fleury E. (2022). κ-carrageenan associated with fructose/glycerol/water LTTM: Toward natural thermosensitive physical gels. ACS Sustain. Chem. Eng..

[44] Liu K., Chen Y.Y., Zha X.Q., Li Q.M., Pan L.H., Luo J.P. (2021). Research progress on polysaccharide/protein hydrogels: Preparation method, functional property and application as delivery systems for bioactive ingredients. Food Res. Int..

[45] Li Y., Zou Y., Que F., Zhang H. (2022). Recent advances in fabrication of edible polymer oleogels for food applications. Curr. Opin. Food Sci..

[46] Pushpamalar J., Meganathan P., Tan H.L., Dahlan N.A., Ooi L.T., Neerooa B.N.H.M., Essa R.Z., Shameli K., Teow S.Y. (2021). Development of a polysaccharide-based hydrogel drug delivery system (DDS): An update. Gels.

[47] Martín-Illana A., Notario-Pérez F., Cazorla-Luna R., Ruiz-Caro R., Bonferoni M.C., Tamayo A., Veiga M.D. (2021). Bigels as drug delivery systems: From their components to their applications. Drug Discov. Today.

[48] Pascuta M.S., Varvara R.A., Teleky B.E., Szabo K., Plamada D., Nemeş S.A., Mitrea L., Martau G.A., Ciont C., Calinoiu L.F. (2022). Polysaccharide-based edible gels as functional ingredients: Characterization, applicability, and human health benefits. Gels.

[49] Shen J., Dai Y., Xia F., Zhang X. (2022). Role of divalent metal ions in the function and application of hydrogels. Prog. Polym. Sci..

[50] Ahmed S., Ikram S. (2016). Chitosan based scaffolds and their applications in wound healing. Achiev. Life Sci..

[51] Younes I., Rinaudo M., Harding D., Sashiwa H. (2015). Chitin and chitosan preparation from marine sources. Structure, properties and applications. Mar. Drugs.

[52] Dash M., Chiellini F., Ottenbrite R.M., Chiellini E. (2011). Chitosan—A versatile semi-synthetic polymer in biomedical applications. Prog. Polym. Sci..

[53] Ghauri Z.H., Islam A., Qadir M.A., Gull N., Haider B., Khan R.U., Riaz T. (2021). Development and evaluation of ph-sensitive biodegradable ternary blended hydrogel films (chitosan/guar gum/PVP) for drug delivery application. Sci. Rep..

[54] Iglesias N., Galbis E., Valencia C., de-Paz M.V., Galbis J.A. (2018). Reversible pH-sensitive chitosan-based hydrogels. influence of dispersion composition on rheological properties and sustained drug delivery. Polymers.

[55] Rinaudo M. (2006). Chitin and chitosan: Properties and applications. Prog. Polym. Sci..

[56] Croisier F., Jérôme C. (2013). Chitosan-Based Biomaterials for Tissue Engineering. Eur. Polym. J..

[57] Phillips D.C. (1967). The hen egg-white lysozyme molecule. Struct. Macromol. Biol. Orig..

[58] Rupley J.A., Gates V. (1967). Studies on the enzymic activity of lysozyme, II. The hydrolysis and transfer reactions of n-a cetylglucosamine oligosaccharides*. Struct. Macromol. Biol. Orig..

[59] Kong M., Chen X.G., Xing K., Park H.J. (2010). Antimicrobial properties of chitosan and mode of action: A state of the art review. Int. J. Food Microbiol..

[60] Sahariah P., Másson M. (2017). Antimicrobial Chitosan and Chitosan Derivatives: A Review of the Structure-Activity Relationship. Biomacromolecules.

[61] Keast D.H., Janmohammad A. (2021). The hemostatic and wound healing effect of chitosan following debridement of chronic ulcers. Wounds.

[62] Khan F., Pham D.T.N., Oloketuyi S.F., Manivasagan P., Oh J., Kim Y.M. (2020). Chitosan and Their Derivatives: Antibiofilm Drugs against Pathogenic Bacteria. Colloids Surf. B Biointerfaces.

[63] Aranaz I., Harris R., Heras A. (2010). Chitosan Amphiphilic Derivatives. Chemistry and Applications. Curr. Org. Chem..

[64] Kim I.Y., Seo S.J., Moon H.S., Yoo M.K., Park I.Y., Kim B.C., Cho C.S. (2008). Chitosan and Its Derivatives for Tissue Engineering Applications. Biotechnol. Adv..

[65] Balan V., Redinciuc V., Tudorachi N., Verestiuc L. (2016). Biotinylated N-palmitoyl chitosan for design of drug loaded self-assembled nanocarriers. Eur. Polym. J..

[66] Rusu A.G., Vulpe R., Popa M.I., Butnaru M., Verestiuc L. Novel semi-interpenetrating polymer networks based on functionalized chitosan and poly(acrylic acid) with potential applications in soft tissue engineering. Proceedings of the 2013 E-Health and Bioengineering Conference (EHB).

[67] Rusu A.G., Tanasa I.A., Popa M.I., Butnaru M., Verestiuc L. Development of novel hydrogels based on citraconyl-chitosan and poly(acrylic acid) as potential wound dressing materials. Proceedings of the 2015 E-Health and Bioengineering Conference, EHB.

[68] Bordenave N., Grelier S., Coma V. (2008). Advances on selective C-6 oxidation of chitosan by TEMPO. Biomacromolecules.

[69] Vold I.M.N., Christensen B.E. (2005). Periodate oxidation of chitosans with different chemical compositions. Carbohydr. Res..

[70] Rusanu A., Tamaş A.I., Vulpe R., Rusu A., Butnaru M., Vereştiuc L. (2017). Biocompatible and biodegradable hydrogels based on chitosan and gelatin with potential applications as wound dressings. J. Nanosci. Nanotechnol..

[71] Vasile C., Pieptu D., Dumitriu R.P., Panzariu A., Profire L. (2013). Chitosan/hyaluronic acid polyelectrolyte complex hydrogels in the management of burn wounds. Rev. Med. Chir. Soc. Med. Nat. Iasi.

[72] Tanasa I.A., Minuti A.E., Ivan F.D., Vasiliu S., Butnaru M., Verestiuc L. Novel Natural-Synthetic Hydrogel Scaffolds with Applications in Skin Tissue Repair and Engineering. Proceedings of the 2017 E-Health and Bioengineering Conference, EHB.

[73] Liu L., Wen H., Rao Z., Zhu C., Liu M., Min L., Fan L., Tao S. (2018). Preparation and characterization of chitosan—Collagen peptide/oxidized konjac glucomannan hydrogel. Int. J. Biol. Macromol..

[74] Chetouani A., Follain N., Marais S., Rihouey C., Elkolli M., Bounekhel M., Benachour D., Le Cerf D. (2017). Physicochemical properties and biological activities of novel blend films using oxidized pectin/chitosan. Int. J. Biol. Macromol..

[75] Baron R.I., Duceac I.A., Morariu S., Bostănaru-Iliescu A.C., Coseri S. (2022). Hemostatic cryogels based on oxidized pullulan/dopamine with potential use as wound dressings. Gels.

[76] Larsson M., Huang W.C., Hsiao M.H., Wang Y.J., Nydén M., Chiou S.H., Liu D.M. (2013). Biomedical applications and colloidal properties of amphiphilically modified chitosan hybrids. Prog. Polym. Sci..

[77] Bhullar N., Rani S., Kumari K., Sud D. (2021). Amphiphilic chitosan/acrylic acid/thiourea based semi-interpenetrating hydrogel: Solvothermal synthesis and evaluation for controlled release of organophosphate pesticide, triazophos. J. Appl. Polym. Sci..

[78] Aliaghaei M., Mirzadeh H., Dashtimoghadam E., Taranejoo S. (2012). Investigation of gelation mechanism of an injectable hydrogel based on chitosan by rheological measurements for a drug delivery application. Soft Matter.

[79] Rusu A.G., Popa M.I., Ibanescu C., Danu M., Verestiuc L. (2016). Tailoring the properties of chitosan-poly(acrylic acid) based hydrogels by hydrophobic monomer incorporation. Mater. Lett..

[80] Rusu A.G., Popa M.I., Lisa G., Vereştiuc L. (2015). Thermal behavior of hydrophobically modified hydrogels using TGA/FTIR/MS analysis technique. Thermochim. Acta.

[81] Liu B., Yang H., Zhu C., Xiao J., Cao H., Simal-Gandara J., Li Y., Fan D., Deng J. (2022). A comprehensive review of food gels: Formation mechanisms, functions, applications, and challenges. Crit. Rev. Food Sci. Nutr.

[82] Sharma S., Parmar A., Mehta S.K., Grumezescu A.M. (2018). Hydrogels: From simple networks to smart materials-advances and applications. Drug Targeting and Stimuli Sensitive Drug Delivery Systems.

[83] Ghorpade V.S., Chen Y. (2020). Preparation of hydrogels based on natural polymers via chemical reaction and cross-Linking. Hydrogels Based on Natural Polymers.

[84] Muir V.G., Burdick J.A. (2021). Chemically Modified Biopolymers for the Formation of Biomedical Hydrogels. Chem. Rev..

[85] Nezhad-Mokhtari P., Ghorbani M., Roshangar L., Rad J.S. (2019). Chemical gelling of hydrogels-based biological macromolecules for tissue engineering: Photo- and enzymatic-crosslinking methods. Int. J. Biol. Macromol..

[86] Simson J.A., Elisseeff J.H., Ma P.X. (2015). Polysaccharide hydrogels for regenerative medicine applications. Hydrogel Scaffolds for Regenerative Medicine.

[87] Franssen O., van Rooijen R.D., de Boer D., Maes R.A.A., Hennink W.E. (1999). Enzymatic degradation of crosslinked dextrans. Macromolecules.

[88] Ferreira L., Figueiredo M.M., Gil M.H., Ramos M.A. (2006). Structural analysis of dextran-based hydrogels obtained chemoenzymatically. J. Biomed. Mater. Res..

[89] Kim S.H., Won C.-Y., Chu C.C. (1999). Synthesis and characterization of dextran-maleic acid based hydrogel. J. Biomed. Mater. Res..

[90] Cadée J.A., de Kerf M., de Groot C.J., den Otter W., Hennink W.E. (1999). Synthesis and characterization of 2-(methacryloyloxy)ethyl-(di)-L-lactate and their application in dextran-based hydrogels. Polymer.

[91] Djabourov M., Bouchemal K., Descamps M. (2016). Polymer Gels, Hydrogels, and Scaffolds – An Overview. Disordered Pharmaceutical Materials.

[92] Rial-Hermida M.I., Rey-Rico A., Blanco-Fernandez B., Carballo-Pedrares N., Byrne E.M., Mano J.F. (2021). Recent Progress on Polysaccharide-Based Hydrogels for Controlled Delivery of Therapeutic Biomolecules. ACS Biomater. Sci. Eng..

[93] Hennink W.E., van Nostrum C.F. (2012). Novel crosslinking methods to design hydrogels. Adv. Drug Deliv. Rev..

[94] Mahinroosta M., Farsangi Z.J., Allahverdi A., Shakoori Z. (2018). Hydrogels as intelligent materials: A brief review of synthesis, properties and applications. Mater. Today Chem..

[95] Zhang Y., Won C.Y., Chu C.C. (2000). Synthesis and characterization of biodegradable hydrophobic-hydrophylic hydrogel networks with a controlled swelling property. J. Polym. Sci. Part A Polym. Chem..

[96] Söderqvist Lindblad M., Sjöberg J., Albertsson A.C., Hartman J., Argyropoulos D.S. (2007). Hydrogels from Polysaccharides for Biomedical Applications. Materials, Chemicals, and Energy from Forest Biomass.

[97] Yuan Z., Wang J., Wang Y., Liu Q., Zhong Y., Wang Y., Li L., Lincoln S.F., Guo X. (2019). Preparation of a poly(acrylic acid) based hydrogel with fast adsorption rate and high adsorption capacity for the removal of cationic dyes. RSC Adv..

[98] Su D., Bai X., He X. (2022). Research progress on hydrogel materials and their antifouling properties. Eur. Polym. J..

[99] Draye J.P., Delaey B., van de Voorde A., van den Bulcke A., de Reu B., Schacht E. (1998). In vitro and in vivo biocompatibility of dextran dialdehyde crosslinked gelatin hydrogel films. Biomaterials.

[100] Vermonden T., Censi R., Hennink W.E. (2012). Hydrogels for Protein Delivery. Chem. Rev..

[101] Nichifor M., Stanciu M.C., Simionescu B.C. (2010). New cationic hydrophilic and amphiphilic polysaccharides synthesized by one pot procedure. Carbohydr. Polym..

[102] Stanciu M.C., Nichifor M., Ailiesei G.L. (2021). Bile salts adsorption on dextran-based hydrogels. Int. J. Biol. Macromol..

[103] Stanciu M.C., Nichifor M., Prisacaru A.-I. (2020). Adsorption of Sodium Cholate on Cationic Dextran Gels: Comparison of Isotherm Binding Models. Mater. Plast..

[104] Stanciu M.C., Nichifor M. (2018). Influence of dextran hydrogel characteristics on adsorption capacity for anionic dyes. Carbohydr. Polym..

[105] Stanciu M.C., Nichifor M. (2019). Adsorption of anionic dyes on a cationic amphiphilic dextran hydrogel: Equilibrium, kinetic, and thermodynamic studies. Colloid Polym. Sci..

[106] Shoukat H., Buksh K., Noreen S., Pervaiz F., Maqbool I. (2021). Hydrogels as potential drug-delivery systems: Network design and applications. Ther. Deliv..

[107] Lu L., Yuan S., Wang J., Shen Y., Deng S., Xie L., Yang Q. (2018). The Formation Mechanism of Hydrogels. Curr. Stem Cell Res. Ther..

[108] Basu A., Kunduru K.R., Doppalapudi S., Domb A.J., Khan W. (2016). Poly(lactic acid) based hydrogels. Adv. Drug Deliv. Rev..

[109] de Jong S.J., van Eerdenbrugh B., van Nostrum C.F., Kettenes-van den Bosch J.J., Hennink W.E. (2001). Physically crosslinked dextran hydrogels by stereocomplex formation of lactic acid oligomers: Degradation and protein release behavior. J. Control. Release.

[110] Wang S., Wang J., Yu J., Wang S. (2016). Effect of fatty acids on functional properties of normal wheat and waxy wheat starches: A structural basis. Food Chem..

[111] Copeland L., Blazek J., Salman H., Tang M.C. (2009). Form and functionality of starch. Food Hydrocoll..

[112] Pérez S., Baldwin P.M., Gallant D.J., BeMiller J., Whistler R. (2009). Structural features of starch granules I. Starch: Chemistry and Technology.

[113] Sasaki T., Matsuki J., Yoza K., Sugiyama J., Maeda H., Shigemune A., Tokuyasu K. (2019). Comparison of textural properties and structure of gels prepared from cooked rice grain under different conditions. Food Sci. Nutr..

[114] Zhu F. (2015). Impact of ultrasound on structure, physicochemical properties, modifications, and applications of starch. Trends Food Sci. Technol..

[115] Ratnayake W.S., Jackson D.S. (2006). A new insight into the gelatinization process of native starches. Carbohydr. Polym..

[116] Liu P., Yu L., Wang X.Y., Li D., Chen L., Li X.X. (2010). Glass transition temperature of starches with different amylose/amylopectin ratios. J. Cereal Sci..

[117] Yu L., Christie G. (2005). Microstructure and mechanical properties of orientated thermoplastic starches. J. Mater. Sci..

[118] Li P., He X., Dhital S., Zhang B., Huang Q. (2017). Structural and physicochemical properties of granular starches after treatment with debranching enzyme. Carbohydr. Polym..

[119] Wang L., Xie B., Shi J., Xue S., Deng Q., Wei Y., Tian B. (2010). Physicochemical properties and structure of starches from Chinese rice cultivars. Food Hydrocoll..

[120] Chen Y., Yang Q., Xu X., Qi L., Dong Z., Luo Z., Lu X., Peng X. (2017). Structural changes of waxy and normal maize starches modified by heat moisture treatment and their relationship with starch digestibility. Carbohydr. Polym..

[121] Huang T.T., Zhou D.N., Jin Z.Y., Xu X.M., Chen H.Q. (2016). Effect of repeated heat-moisture treatments on digestibility, physicochemical and structural properties of sweet potato starch. Food Hydrocoll..

[122] Lopez-Rubio A., Flanagan B.M., Gilbert E.P., Gidley M.J. (2008). A novel approach for calculating starch crystallinity and its correlation with double helix content: A combined XRD and NMR study. Biopolymers.

[123] Xu X., Chen Y., Luo Z., Lu X. (2019). Different variations in structures of A- and B-type starches subjected to microwave treatment and their relationships with digestibility. LWT.

[124] Cai C., Wei C. (2013). In situ observation of crystallinity disruption patterns during starch gelatinization. Carbohydr. Polym..

[125] Yang Z., Gu Q., Hemar Y. (2013). In situ study of maize starch gelatinization under ultra-high hydrostatic pressure using X-ray diffraction. Carbohydr. Polym..

[126] Dobosz A., Sikora M., Krystyjan M., Tomasik P., Lach R., Borczak B., Berski W., Lukasiewicz M. (2019). Short-and long-term retrogradation of potato starches with varying amylose content. J. Sci. Food Agric..

[127] Wang S., Li C., Copeland L., Niu Q., Wang S. (2015). Starch retrogradation: A comprehensive review. Compr. Rev. Food Sci. Food Saf..

[128] Varma C.A.K., Panpalia S.G., Kumar K.J. (2014). Physicochemical and release kinetics of natural and retrograded starch of Indian palmyrah shoots. Int. J. Biol. Macromol..

[129] Bashir K., Aggarwal M. (2019). Physicochemical, structural and functional properties of native and irradiated starch: A review. J. Food Sci. Technol..

[130] Jyothi A.N., Sajeev M.S., Sreekumar J.N. (2010). Hydrothermal modifications of tropical tuber starches. 1. Effect of heat-moisture treatment on the physicochemical, rheological and gelatinization characteristics. Starch.

[131] Gunaratne A., Hoover R. (2002). Effect of heat–moisture treatment on the structure and physicochemical properties of tuber and root starches. Carbohydr. Polym..

[132] Tan X., Li X., Chen L., Xie F., Li L., Huang J. (2017). Effect of heat-moisture treatment on multi-scale structures and physicochemical properties of breadfruit starch. Carbohydr. Polym..

[133] Bian L., Chung H.J. (2016). Molecular structure and physicochemical properties of starch isolated from hydrothermally treated brown rice flour. Food Hydrocoll..

[134] Sui Z., Yao T., Zhao Y., Ye X., Kong X., Ai L. (2015). Effects of heat–moisture treatment reaction conditions on the physicochemical and structural properties of maize starch: Moisture and length of heating. Food Chem..

[135] Hoover R. (2010). The impact of heat-moisture treatment on molecular structures and properties of starches isolated from different botanical sources. Crit. Rev. Food Sci. Nutr..

[136] Klein B., Pinto V.Z., Vanier N.L., da Rosa Zavareze E., Colussi R., do Evangelho J.A., Gutkoski L.C., Dias A.R.G. (2013). Effect of single and dual heat-moisture treatments on properties of rice, cassava, and pinhão starches. Carbohydr. Polym..

[137] da Rosa Zavareze E., Stork C.R., Castro L.A.S., Schirmer M.A., Dias A.R.G. (2010). Effect of heat-moisture treatment on rice starch of varying amylose content. Food Chem..

[138] Pepe L.S., Moraes J., Albano K.M., Telis V.R., Franco C.M. (2016). Effect of heat-moisture treatment on the structural, physicochemical, and rheological characteristics of arrowroot starch. Food Sci. Technol. Int..

[139] Jiranuntakul W., Puttanlek C., Rungsardthong V., Puncha-Arnon S., Uttapap D. (2011). Microstructural and physicochemical properties of heat-moisture treated waxy and normal starches. J. Food Eng..

[140] da Rosa Zavareze E., Dias A.R.G. (2011). Impact of heat-moisture treatment and annealing in starches: A review. Carbohydr. Polym..

[141] Sui Z., Shah A., BeMiller J.N. (2011). Crosslinked and stabilized in-kernel heat-moisture-treated and temperature-cycled normal maize starch and effects of reaction conditions on starch properties. Carbohydr. Polym..

[142] Varatharajan V., Hoover R., Li J., Vasanthan T., Nantanga K.K.M., Seetharaman K., Liu Q., Donner E., Jaiswal S., Chibbar R.N. (2011). Impact of structural changes due to heat-moisture treatment at different temperatures on the susceptibility of normal and waxy potato starches towards hydrolysis by porcine pancreatic alpha amylase. Food Res. Int..

[143] Xu F., Zhang L., Liu W., Liu Q., Wang F., Zhang H., Hu H., Blecker C. (2021). Physicochemical and Structural Characterization of Potato Starch with Different Degrees of Gelatinization. Foods.

[144] Ambigaipalan P., Hoover R., Donner E., Liu Q. (2014). Starch chain interactions within the amorphous and crystalline domains of pulse starches during heat-moisture treatment at different temperatures and their impact on physicochemical properties. Food Chem..

[145] Vermeylen R., Goderis B., Delcour J.A. (2006). An X-ray study of hydrothermally treated potato starch. Carbohydr. Polym..

[146] Singh G.D., Bawa A.S., Riar C.S., Saxena D.C. (2009). Influence of heat moisture treatment and acid modifications on physicochemical, rheological, thermal and morphological characteristics of Indian water chestnut (*Trapa natans)* starch and its applications in biodegradable films. Starch.

[147] Alvani K., Qi X., Tester R.F. (2012). Gelatinization properties of native and annealed potato starches. Starch.

[148] Zhong Y., Xiang X., Zhao J., Wang X., Chen R., Xu J., Luo S., Wu J., Liu C. (2020). Microwave pretreatment promotes the annealing modification of rice starch. Food Chem..

[149] Yadav B.S., Guleria P., Yadav R.B. (2013). Hydrothermal modification of Indian water chestnut starch: Influence of heat-moisture treatment and annealing on the physicochemical, gelatinization and pasting characteristics. LWT.

[150] Egharevba H.O., Emeje M.O. (2020). Chemical Properties of Starch and Its Application in the Food Industry. Chemical Properties of Starch.

[151] Lee S.J., Hong J.Y., Lee E.J., Chung H.J., Lima S.T. (2015). Impact of single and dual modifications on physicochemical properties of *japonica* and *indica* rice starches. Carbohydr. Polym..

[152] Wang H., Zhu Q., Wu T., Zhang M. (2020). Glass transition temperature, rheological, and gelatinization properties of high amylose corn starch and waxy cassava starch blends. J. Food Process. Preserv..

[153] Lee E.C., Lee J., Chung H.J., Park E.Y. (2021). Impregnation of normal maize starch granules with ionic hydrocolloids by alkaline dry heating. Food Hydrocoll..

[154] Zhao Q., Tian H., Chen L., Zeng M., Qin F., Wang Z., He Z., Chen J. (2021). Interactions between soluble soybean polysaccharide and starch during the gelatinization and retrogradation: Effects of selected starch varieties. Food Hydrocoll..

[155] Luo Y., Shen M., Li E., Xiao Y., Wen H., Ren Y., Xie J. (2020). Effect of *Mesona chinensis* polysaccharide on pasting, rheo-logical and structural properties of corn starches varying in amylose contents. Carbohydr. Polym..

[156] Xie F., Zhang H., Xia Y., Ai L. (2020). Effects of tamarind seed polysaccharide on gelatinization, rheological, and structural properties of corn starch with different amylose/amylopectin ratios. Food Hydrocoll..

[157] Yan W., Yin L., Zhang M., Zhang M., Jia X. (2021). Gelatinization, Retrogradation and Gel Properties of Wheat Starch–Wheat Bran Arabinoxylan Complexes. Gels.

[158] Pongsawatmanit R., Temsiripong T., Suwonsichon T. (2007). Thermal and rheological properties of tapioca starch and xyloglucan mixtures in the presence of sucrose. Food Res. Int..

[159] Sun Q., Dai L., Nan C., Xiong L. (2014). Effect of heat moisture treatment on physicochemical and morphological properties of wheat starch and xylitol mixture. Food Chem..

[160] Buchtová N., Budtova T. (2016). Cellulose aero-, cryo- and xerogels: Towards understanding of morphology control. Cellulose.

[161] White R.J., Brun N., Budarin V.L., Clark J.H., Titirici M.M. (2014). Always look on the “light” side of life: Sustainable carbon aerogels. ChemSusChem.

[162] Cheng H., Gu B., Pennefather M.P., Nguyen T.X., Phan-Thien N., Duong H.M. (2017). Cotton aerogels and cotton-cellulose aerogels from environmental waste for oil spillage cleanup. Mater. Des..

[163] Kaya M. (2017). Super absorbent, light, and highly flame-retardant cellulose-based aerogel crosslinked with citric acid. J. Appl. Polym. Sci..

[164] Bao M.X., Xu S., Wang X., Sun R. (2016). Porous cellulose aerogels with high mechanical performance and their absorption behaviors. BioResources.

[165] Feng J., Nguyen S.T., Fan Z., Duong H.M. (2015). Advanced fabrication and oil absorption properties of super-hydrophobic recycled cellulose aerogels. Chem. Eng. J..

[166] Yang X., Cranston E.D. (2014). Chemically cross-linked cellulose nanocrystal aerogels with shape recovery and superabsorbent properties. Chem. Mater..

[167] Granström M., née Pääkkö M.K., Jin H., Kolehmainen E., Kilpeläinen I., Ikkala O. (2011). Highly water repellent aerogels based on cellulose stearoyl esters. Polym. Chem..

[168] Jin H., Kettunen M., Laiho A., Pynnönen H., Paltakari J., Marmur A., Ikkala O., Ras R.H.A. (2011). Superhydrophobic and superoleophobic nanocellulose aerogel membranes as bioinspired cargo carriers on water and oil. Langmuir.

[169] Aulin C., Netrval J., Wågberg L., Lindström T. (2010). Aerogels from nanofibrillated cellulose with tunable oleophobicity. Soft Matter.

[170] Nguyen S.T., Feng J., Ng S.K., Wong J.P.W., Tan V.B.C., Duong H.M. (2014). Advanced thermal insulation and absorption properties of recycled cellulose aerogels. Colloids Surf. A Physicochem. Eng. Asp..

[171] Hayase G., Kanamori K., Abe K., Yano H., Maeno A., Kaji H., Nakanishi K. (2014). Polymethylsilsesquioxane–cellulose nanofiber biocomposite aerogels with high thermal insulation, bendability, and superhydrophobicity. ACS Appl. Mater. Interfaces.

[172] Kobayashi Y., Saito T., Isogai A. (2014). Aerogels with 3D ordered nanofiber skeletons of liquid-crystalline nanocellulose derivatives as tough and transparent insulators. Angew. Chem. Int. Ed..

[173] Uddin K.M.A., Orelma H., Mohammadi P., Borghei M., Laine J., Linder M., Rojas O.J. (2017). Retention of lysozyme activity by physical immobilization in nanocellulose aerogels and antibacterial effects. Cellulose.

[174] Zhang C., Zhai T., Turng L.S. (2017). Aerogel microspheres based on cellulose nanofibrils as potential cell culture scaffolds. Cellulose.

[175] Wang C., Liu W., Cao H., Jia L., Liu P. (2022). Cellulose nanofibers aerogels functionalized with AgO: Preparation, characterization and antibacterial activity. Int. J. Biol. Macromol..

[176] Zhao J., Lu C., He X., Zhang X., Zhang W., Zhang X. (2015). Polyethylenimine-grafted cellulose nanofibril aerogels as versatile vehicles for drug delivery. ACS Appl. Mater. Interfaces.

[177] Henschen J., Illergård J., Larsson P.A., Ek M., Wågberg L. (2016). Contact-active antibacterial aerogels from cellulose nanofibrils. Colloids Surf. B Biointerfaces.

[178] Cai H., Sharma S., Liu W., Mu W., Liu W., Zhang X., Deng Y. (2014). Aerogel microspheres from natural cellulose nanofibrils and their application as cell culture scaffold. Biomacromolecules.

[179] Wang H., Gong Y., Wang Y. (2014). Cellulose-based hydrophobic carbon aerogels as versatile and superior adsorbents for sewage treatment. RSC Adv..

[180] Wu Z.-Y., Li C., Liang H.-W., Chen J.-F., Yu S.-H. (2013). Ultralight, flexible, and fire-resistant carbon nanofiber aerogels from bacterial cellulose. Angew. Chem. Int. Ed..

[181] Schestakow M., Muench F., Reimuth C., Ratke L., Ensinger W. (2016). Electroless synthesis of cellulose-metal aerogel composites. Appl. Phys. Lett..

[182] Han Y., Zhang X., Wu X., Lu C. (2015). Flame retardant, heat insulating cellulose aerogels from waste cotton fabrics by in situ formation of magnesium hydroxide nanoparticles in cellulose gel nanostructures. ACS Sustain. Chem. Eng..

[183] Cai J., Kimura S., Wada M., Kuga S. (2009). Nanoporous cellulose as metal nanoparticles support. Biomacromolecules.

[184] Li Z., Lin Z. (2021). Recent advances in polysaccharide-based hydrogels for synthesis and applications. Aggregate.

[185] Guo Y., Bae J., Fang Z., Li P., Zhao F., Yu G. (2020). Hydrogels and hydrogel-derived materials for energy and water sustainability. Chem. Rev..

[186] Kabir S.M.F., Sikdar P.P., Haque B., Bhuiyan M.A.R., Ali A., Islam M.N. (2018). Cellulose-based hydrogel materials: Chemistry, properties and their prospective applications. Prog. Biomater..

[187] Chang C., Zhang L. (2011). Cellulose-based hydrogels: Present status and application prospects. Carbohydr. Polym..

[188] Shogren R.L., Peterson S.C., Evans K.O., Kenar J.A. (2011). Preparation and characterization of cellulose gels from corn cobs. Carbohydr. Polym..

[189] Nakasone K., Kobayashi T. (2016). Effect of pre-treatment of sugarcane bagasse on the cellulose solution and application for the cellulose hydrogel films. Polym. Adv. Technol..

[190] Nakasone K., Kobayashi T. (2016). Cytocompatible cellulose hydrogels containing trace lignin. Mater. Sci. Eng. C.

[191] Nakasone K., Ikematsu S., Kobayashi T. (2016). Biocompatibility evaluation of cellulose hydrogel film regenerated from sugarcane bagasse waste and its in vivo behavior in mice. Ind. Eng. Chem. Res..

[192] Kobayashi T., Thakur V.K., Thakur M.K. (2018). Cellulose hydrogels: Fabrication, properties, and their application to biocompatible and tissue engineering. Hydrogels, Gels Horizons: From Science to Smart Materials.

[193] Long L.-Y., Weng Y.-X., Wang Y.-Z. (2018). Cellulose aerogels: Synthesis, applications, and prospects. Polymers.

[194] Duceac I.A., Tanasă F., Coşeri S. (2022). Selective oxidation of cellulose—A multitask platform with significant environmental impact. Materials.

[195] Yamasaki S., Sakuma W., Yasui H., Daicho K., Saito T., Fujisawa S., Isogai A., Kanamori K. (2019). Nanocellulose xerogels with high porosities and large specific surface areas. Front. Chem..

[196] Paladini G., Venuti V., Crupi V., Majolino D., Fiorati A., Punta C. (2020). FTIR-ATR analysis of the H-bond network of water in branched polyethyleneimine/TEMPO-oxidized cellulose nano-fiber xerogels. Cellulose.

[197] Rbihi S., Laallam L., Sajieddine M., Jouaiti A. (2019). Characterization and thermal conductivity of cellulose based composite xerogels. Heliyon.

[198] Pottathara Y.B., Bobnar V., Finšgar M., Grohens Y., Thomas S., Kokol V. (2018). Cellulose nanofibrils-reduced graphene oxide xerogels and cryogels for dielectric and electrochemical storage applications. Polymer.

[199] Marr P.C., Marr A.C. (2016). Ionic liquid gel materials: Applications in green and sustainable chemistry. Green Chem..

[200] Hopson C., Villar-Chavero M.M., Domínguez J.C., Alonso M.V., Oliet M., Rodriguez F. (2021). Cellulose ionogels, a perspective of the last decade: A review. Carbohydr. Polym..

[201] Wang Z., Liu J., Zhang J., Hao S., Duan X., Song H., Zhang J. (2020). Novel chemically cross-linked chitosan-cellulose based ionogel with self-healability, high ionic conductivity, and high thermo-mechanical stability. Cellulose.

[202] Teacă C.-A., Stanciu M.-C., Tanasă F., Nechifor M., Inamuddin, Abdullah M.A. (2020). Ionic liquids for enhanced enzymatic saccharification of cellulose-based materials. Nanotechnology Based Industrial Applications of Ionic Liquids.

[203] Takada A., Kadokawa J. (2022). Preparation of cellulosic soft and composite materials using ionic liquid media and ion gels. Cellulose.

[204] Kadokawa J., Sarker M.I., Liu L.S., Yadav M.P., Yosief H.O., Hussain S.A. (2021). Preparation of cellulose-based soft and composite materials through dissolution and gelation with ionic liquids. Conversion of Renewable Biomass into Bioproducts.

[205] Idenoue S., Oga Y., Hashimoto D., Yamamoto K., Kadokawa J. (2019). Preparation of reswellable amorphous porous celluloses through hydrogelation from ionic liquid solutions. Materials.

[206] Zhang J., Wu J., Yu J., Zhang X., He J., Zhang J. (2017). Application of ionic liquids for dissolving cellulose and fabricating cellulose-based materials: State of the art and future trends. Mater. Chem. Front..

[207] Takada A., Kadokawa J. (2015). Fabrication and characterization of polysaccharide ion gels with ionic liquids and their further conversion into value-added sustainable materials. Biomolecules.

[208] Teacă C.-A., Bodîrlău R., Spiridon I. (2011). Dissolution of natural polymers in ionic liquid. Rev. Roum. Chim..

[209] Gupta K.M., Hu Z., Jiang J. (2013). Cellulose regeneration from a cellulose/ionic liquid mixture: The role of anti-solvents. RSC Adv..

[210] O’Connor R.T., DuPre E.F., Mitcham D. (1958). Application of infrared absorption spectroscopy to investigations of cotton and modified cottons. Part 1: Physical and crystalline modifications and oxidation. Text. Res. J..

[211] Nelson M.L., O’Connor R.T. (1964). Relation of certain infrared bands to cellulose crystallinity and crystal latticed type. Part I. Spectra of lattice types I, II, III and of amorphous cellulose. J. Appl. Polym. Sci..

[212] Oh S.Y., Dong I.Y., Shin Y., Hwan C.K., Hak Y.K., Yong S.C., Won H.P., Ji H.Y. (2005). Crystalline structure analysis of cellulose treated with sodium hydroxide and carbon dioxide by means of X-ray diffraction and FTIR spectroscopy. Carbohydr. Res..

[213] Gupta K.M., Hu Z., Jiang J. (2013). Molecular insight into cellulose regeneration from a cellulose/ionic liquid mixture: Effects of water concentration and temperature. RSC Adv..

[214] Ibrahim N.A., Nada A.A., Eid B.M., Thakur V.K., Thakur M.K. (2018). Polysaccharide-based polymer gels and their potential applications. Polymer Gels, Gels Horizons: From Science to Smart Materials.

[215] Agulhon P., Robitzer M., Habas J.P., Quignard F. (2014). Influence of both cation and alginate nature on the rheological behavior of transition metal alginate gels. Carbohydr. Polym..

[216] Brus J., Urbanova M., Czernek J., Pavelkova M., Kubova K., Vyslouzil J., Abbrent S., Konefal R., Horský J., Vetchy D. (2017). Structure and dynamics of alginate gels cross-linked by polyvalent ions probed via solid state NMR spectroscopy. Biomacromolecules.

[217] Wang M., Chen L., Zhang Z. (2021). Potential applications of alginate oligosaccharides for biomedicine—A mini review. Carbohydr. Polym..

[218] Pawar S.N., Edgar K.J. (2012). Alginate derivatization: A review of chemistry, properties and applications. Biomaterials.

[219] Lee K.Y., Mooney D.J. (2012). Alginate: Properties and biomedical applications. Prog. Polym. Sci..

[220] Vicini S., Castellano M., Mauri M., Marsano E. (2015). Gelling process for sodium alginate: New technical approach by using calcium rich micro-spheres. Carbohydr. Polym..

[221] Caccavo D., Ström A., Larsson A., Lamberti G. (2016). Modeling capillary formation in calcium and copper alginate gels. Mater. Sci. Eng. C.

[222] Augst A.D., Kong H.J., Mooney D.J. (2006). Alginate hydrogels as biomaterials. Macromol. Biosci..

[223] Chan G., Mooney D.J. (2013). Ca^2+^ released from calcium alginate gels can promote inflammatory responses in vitro and in vivo. Acta Biomater..

[224] Bi D., Lai Q., Cai N., Li T., Zhang Y., Han Q., Peng Y., Xu H., Lu J., Bao W. (2018). Elucidation of the molecular-mechanisms and in vivo evaluation of the anti-inflammatory effect of alginate-derived seleno-polymannuronate. J. Agric. Food Chem..

[225] Popescu I., Turtoi M., Suflet D.M., Dinu M.V., Darie-Nita R.N., Anghelache M., Calin M., Constantin M. (2021). Alginate/poloxamer hydrogel obtained by thiol-acrylate photopolymerization for the alleviation of the inflammatory response of human keratinocytes. Int. J. Biol. Macromol..

[226] Sahoo D.R., Biswal T. (2021). Alginate and its application to tissue engineering. Springer Nat. Appl. Sci..

[227] Zhong H., Gao X., Cheng C., Liu C., Wang Q., Han X. (2020). The structural characteristics of seaweed polysaccharides and their application in gel drug delivery systems. Mar. Drugs.

[228] Yang J.S., Xie Y.J., He W. (2011). Research progress on chemical modification of alginate: A review. Carbohydr. Polym..

[229] Van Vlierberghe S., Dubruel P., Schacht E. (2011). Biopolymer-based hydrogels as scaffolds for tissue engineering applications: A review. Biomacromolecules.

[230] Zhang J., Daubert C.R., Foegeding E.A. (2007). A proposed strain-hardening mechanism for alginate gels. J. Food Eng..

[231] Stokke B.T., Draget K.I., Smidsrod O., Yuguchi Y., Urakawa H., Kajiwara K. (2000). Small-angle X-ray scattering and rheological characterization of alginate gels. 1. Ca-alginate gels. Macromolecules.

[232] Draget K.I., Stokke B.T., Yuguchi Y., Urakawa H., Kajiwara K. (2003). Small-angle X-ray scattering and rheological characterization of alginate gels. 3. Alginic acid gels. Biomacromolecules.

[233] Agulhon P., Robitzer M., David L., Quignard F. (2012). Structural regime identification in ionotropic alginate gels: Influence of the cation nature and alginate structure. Biomacromolecules.

[234] Hermansson E., Schuster E., Lindgren L., Altskär A., Ström A. (2016). Impact of solvent quality on the network strength and structure of alginate gels. Carbohydr. Polym..

[235] Enev V., Sedláček P., Řihák M., Kalina M., Pekař M. (2022). IR-Supported Thermogravimetric Analysis of Water in Hydrogels. Front. Mater..

[236] Chan A.W., Whitney R.A., Neufeld R.J. (2009). Semi-synthesis of a controlled stimuli-responsive alginate hydrogel. Biomacromolecules.

[237] Li A., Guo C., Li X., Li P., Yang X., Guo Y. (2021). Gelation mechanism and physical properties of glucono-δ-lactone induced alginate sodium/casein composite gels. Food Hydrocoll..

[238] Li A., Gong T., Yang X., Guo Y. (2020). Interpenetrating network gels with tunable physical properties: Glucono-δ-lactone induced gelation of mixed Alg/gellan sol systems. Int. J. Biol. Macromol..

[239] Ehterami A., Salehi M., Farzamfar S., Samadian H., Vaez A., Sahrapeyma H., Ghorbani S. (2020). A promising wound dressing based on alginate hydrogels containing vitamin D3 cross-linked by calcium carbonate/d-glucono-δ-lactone. Biomed. Eng. Lett..

[240] Lee K.Y., Bouhadir K.H., Mooney D.J. (2004). Controlled degradation of hydrogels using multi-functional cross-linking molecules. Biomaterials.

[241] Jeon O., Bouhadir K.H., Mansour J.M., Alsberg E. (2009). Photocrosslinked alginate hydrogels with tunable biodegradation rates and mechanical properties. Biomaterials.

[242] Jeon O., Powell C., Ahmed S.M., Alsberg E. (2010). Biodegradable, photocrosslinked alginate hydrogels with independently tailorable physical properties and cell adhesivity. Tissue Eng. Part A.

[243] Zhao D., Wang X., Tie C., Cheng B., Yang S., Sun Z., Yin M., Li X., Yin M. (2021). Bio-functional strontium-containing photocrosslinked alginate hydrogels for promoting the osteogenic behaviors. Mater. Sci. Eng. C.

[244] Gurikov P., Smirnova I. (2018). Non-conventional methods for gelation of alginate. Gels.

[245] Zhao Y., Shen W., Chen Z., Wu T. (2016). Freeze-thaw induced gelation of alginates. Carbohydr. Polym..

[246] Tkalec G., Kranvogl R., Perva Uzunalic A., Knez Ž., Novak Z. (2016). Optimisation of critical parameters during alginate aerogels production. J. Non-Cryst. Solids.

[247] Guillen G.R., Pan Y., Li M., Hoek E.M.V. (2011). Preparation and Characterization of Membranes Formed by Nonsolvent Induced Phase Separation: A Review. Ind. Eng. Chem. Res..

[248] Pérez-Madrigal M.M., Torras J., Casanovas J., Häring M., Aleman C., Díaz Díaz D. (2017). A paradigm shift for preparing versatile M^2+^-free gels from unmodified sodium alginate. Biomacromolecules.

[249] Martins M., Barros A.A., Quraishi S., Gurikov P., Raman S.P., Smirnova I., Duarte A.R.C., Reis R.L. (2015). Preparation of macroporous alginate-based aerogels for biomedical applications. J. Supercrit. Fluids.

[250] Quraishi S., Martins M., Barros A.A., Gurikov P., Raman S.P., Smirnova I., Duarte A.R.C., Reis R.L. (2015). Novel non-cytotoxic alginate-lignin hybrid aerogels as scaffolds for tissue engineering. J. Supercrit. Fluids.

[251] Liu X., Liu H., Qu X., Lei M., Zhang C., Hong H., Payne G.F., Liu C. (2017). Electrical signals triggered controllable formation of calcium-alginate film for wound treatment. J. Mater. Sci. Mater. Med..

[252] Bruchet M., Melman A. (2015). Fabrication of patterned calcium cross-linked alginate hydrogel films and coatings through reductive cation exchange. Carbohydr. Polym..

[253] Hassan R.M. (2021). Novel synthesis of natural cation exchange resin by crosslinking the sodium alginate as a natural polymer with 1, 6-hexamethylene diisocyanate in inert solvents: Characteristics and applications. Int. J. Biol. Macromol..

